# Lysosomal expansion compartments mediate zinc and copper homeostasis in *Caenorhabditis elegans*

**DOI:** 10.1093/mtomcs/mfag030

**Published:** 2026-10-07

**Authors:** Joy R Armendariz, Sean Teng, Camdyn Rakow, Raquel Herrera, Samuel Herrera, Moshe T Gordon, Si Chen, Stefan Vogt, Hanwenheng Liu, Makena Jarvis, Katryna Reese, Aidan T Pezacki, Christopher J Chang, Byung-Eun Kim, Daniel L Schneider, Adelita D Mendoza, Kerry Kornfeld

**Affiliations:** Department of Biochemistry, University of Colorado, Boulder, CO 80303, United States; Department of Developmental Biology, Washington University School of Medicine, Saint Louis, MO 63110, United States; Department of Biochemistry, University of Colorado, Boulder, CO 80303, United States; Department of Developmental Biology, Washington University School of Medicine, Saint Louis, MO 63110, United States; Department of Developmental Biology, Washington University School of Medicine, Saint Louis, MO 63110, United States; Department of Biochemistry, University of Colorado, Boulder, CO 80303, United States; X-ray Science Division, Advanced Photon Source, Argonne National Laboratory, Lemont, IL 60439, United States; X-ray Science Division, Advanced Photon Source, Argonne National Laboratory, Lemont, IL 60439, United States; Department of Developmental Biology, Washington University School of Medicine, Saint Louis, MO 63110, United States; Department of Biochemistry, University of Colorado, Boulder, CO 80303, United States; Department of Biochemistry, University of Colorado, Boulder, CO 80303, United States; Department of Chemistry, Princeton University, Princeton, NJ 08544, United States; Department of Chemistry, University of California at Berkeley, Berkeley, CA 94720, United States; Department of Chemistry, Princeton University, Princeton, NJ 08544, United States; Department of Chemistry, University of California at Berkeley, Berkeley, CA 94720, United States; Department of Animal and Avian Sciences, University of Maryland, College Park, MD 20742, United States; Department of Developmental Biology, Washington University School of Medicine, Saint Louis, MO 63110, United States; Department of Biochemistry, University of Colorado, Boulder, CO 80303, United States; Department of Developmental Biology, Washington University School of Medicine, Saint Louis, MO 63110, United States; Department of Developmental Biology, Washington University School of Medicine, Saint Louis, MO 63110, United States

## Abstract

Zinc is an essential transition metal that participates in many biological processes. In *Caenorhabditis elegans*, excess zinc is stored in lysosomes in intestinal cells; this process involves increasing the expression of the zinc transporter CDF-2 and remodeling of lysosomes characterized by an increase in the volume of the expansion compartment. To determine if this is a more general property, we investigated other metals. Here we report that lysosomes are remodeled in response to excess copper, manganese, and cadmium, with each metal causing an increase in the volume of the expansion compartment. Mutants with a reduced number of lysosomes were hypersensitive to growth retardation caused by excess copper and manganese, suggesting metal toxicity is prevented by metal sequestration in lysosomes. We developed a method to analyze isolated lysosomes by X-ray Fluorescence Microscopy; zinc, copper, and manganese are detectable in the lumen of lysosomes, and the concentration of zinc in the lysosomes increased substantially in worms exposed to excess dietary zinc. To further analyze copper, we examined localization of CUA-1.1, a copper transporter that moves copper into the lumen of lysosomes. Like the zinc transporter CDF-2, CUA-1.1 localizes to both the acidified and expansion compartments in excess copper. These results indicate that the same intestinal lysosomes store both zinc and copper. Furthermore, lysosome remodeling characterized by an increase in volume of the expansion compartment is not specific to excess zinc but is a more general phenomenon during metal storage in lysosomes.

## Graphical Abstract

**Graphical Abstract:**
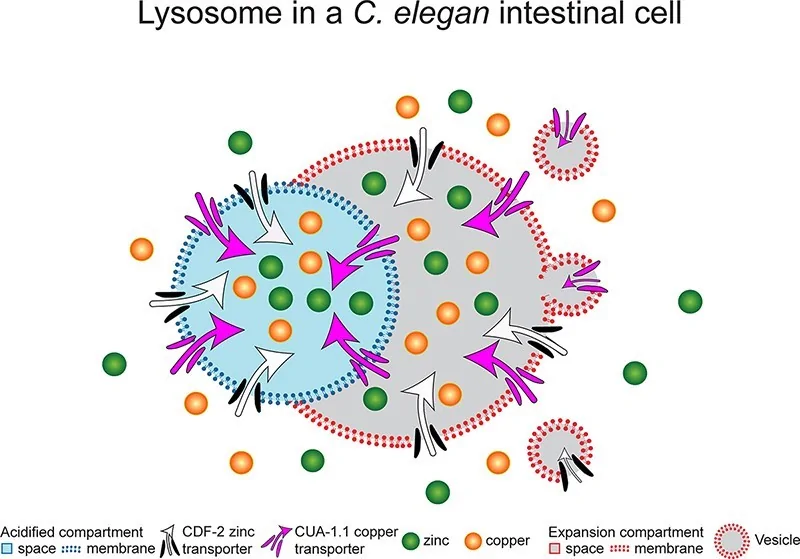

## Introduction

Zinc is a transition metal that all organisms require for survival [1,2]. Zinc is predicted to bind ∼10% of the human proteome, corresponding to ∼3000 proteins [3]. Protein bound zinc plays essential roles in enzyme catalysis [3] and protein structure [4], whereas labile zinc functions as a signal transduction molecule [5]. Given this widespread requirement, organisms have evolved sophisticated zinc homeostasis mechanisms to tightly regulate intracellular zinc levels. Dysregulation of zinc results in either zinc deficiency or zinc toxicity. In humans, inadequate dietary zinc or inborn errors of zinc metabolism lead to pathologies such as acrodermatitis enteropathica [6] and neurodegeneration [7, 8].

Zinc homeostasis is partly mediated by transmembrane proteins that transport zinc across membranes. Members of the SLC30A/ZNT/CDF protein family transport zinc from the cytosol to extracellular spaces or luminal spaces within cells [9, 10]. Members of the SLC39A/Zrt, Irt-like (ZIP) protein family transport zinc into the cytosol from intracellular compartments or extracellular spaces [9, 10]. Metallothioneins are small molecular weight proteins localized to the cytosol that can bind multiple atoms of metals such as zinc, copper, and cadmium [10–12]. These sulfhydryl-rich proteins serve as a buffer for excess metals.

*Caenorhabditis elegans* has been used to study zinc homeostasis because this tractable model organism offers powerful genetic analysis, live fluorescence imaging enabled by a translucent body, and conservation with humans (~30% of *C. elegans* genes are orthologous to human genes) [13]. In *C. elegans*, high- and low-zinc homeostasis pathways have been identified and are thought to function in parallel [14, 15]. In excess zinc conditions, the high zinc sensor HIZR-1, a nuclear receptor transcription factor, senses elevated cytosolic zinc levels by binding zinc at its ligand-binding domain. Zinc bound HIZR-1 translocates to the nucleus, where the DNA-binding domain interacts with high specificity and affinity with the High Zinc Activation (HZA) enhancer element [16, 17]. This leads to the upregulation of multiple genes that are active during high zinc homeostasis, including *cdf-2*. The CDF-2 protein transports zinc from the cytosol into the lumen of intestinal lysosomes, resulting in zinc storage [18, 19]. The low zinc homeostasis pathway is less defined but involves the activation of the Low Zinc Activation (LZA) enhancer element, resulting in the upregulation of low zinc homeostasis genes, including *zipt-2.3* [15]. The ZIPT-2.3 protein transports zinc from the lumen of lysosomes into the cytosol [20]. The CDF-2 and ZIPT-2.3 proteins are reciprocally regulated by zinc concentration at the level of transcription and protein abundance [20].

Lysosomes in intestinal cells of *C. elegans* contain two compartments—an acidified compartment, and a dynamic expansion compartment that is not acidic. Changes in zinc concentration lead to morphological restructuring in lysosomes [19, 20]. In zinc-replete conditions, the acidified compartment predominates, whereas the expansion compartment is small. In zinc-deficient conditions, the expansion compartment is moderately increased in volume, while in zinc-excess conditions, the expansion compartment is dramatically increased in volume [20]. In each case, CDF-2 localizes to the membrane of the acidified compartment and the expansion compartment, whereas ZIPT-2.3 localizes to the membrane of the acidified compartment but not the expansion compartment [20]. This morphological remodeling is predicted to facilitate a change in the ratio of CDF-2 and ZIPT-2.3 on the membrane of the lysosome, resulting in a net flow of zinc into lysosomes in zinc-excess conditions or a net outflow of zinc into the cytosol in zinc-deficient conditions.

The findings that zinc is stored in lysosomes and excess zinc induces morphological remodeling led us to ask if other transition metals are also stored in lysosomes and induce lysosome remodeling. Copper and manganese are essential metals necessary for a wide range of cellular processes such as cell fate, mitochondrial function, immune function, digestion, and metabolism [21–23]. Cadmium is not an essential metal but is a potent toxin—it is in the same group as zinc on the periodic table and is predicted to displace zinc from important binding sites in proteins [24–26]. Like zinc, copper also requires tight homeostatic regulation; dysregulation of copper leads to oxidative cellular stress [27, 28] and human pathologies such as Wilson’s and Menkes diseases [29, 30]. Lysosomes have been linked to copper homeostasis and are emerging as an important site for copper trafficking. In *C. elegans*, intestinal cells are a key site for copper homeostasis; in replete copper conditions, the copper transporter CUA-1.1 (ATP7A/B) localizes to the basolateral membrane where it transports copper into the pseudocoelom [31]. However, exposure to excess copper causes re-localization of CUA-1.1 to the membrane of lysosomes where it transports copper into the lysosomal lumen [31]. This contrasts with the zinc transporter CDF-2, which constitutively localizes to lysosomes and is not known to function elsewhere. Regulation of *cua-1.1* also contrasts with *cdf-2*, because exposure to copper leads to downregulation of *cua-1.1* mRNA, while exposure to zinc leads to upregulation of *cdf-2* mRNA [18, 31]. Although CUA-1.1 localizes to lysosomes in excess copper, its relationship to the expansion compartment remains unknown.

Here we report that lysosomes are remodeled in response to excess copper, manganese, and cadmium, with each condition causing an increase in the volume of the expansion compartment. Mutants with a reduced number of lysosomes were hypersensitive to growth retardation caused by excess copper and manganese, suggesting these metals are sequestered in lysosomes. To quantify the metal content of intestinal lysosomes, we developed a new method to analyze isolated lysosomes by X-ray Fluorescence Microscopy (XFM). Zinc, copper, and manganese were detectable in the lumen of lysosomes. To probe the mechanism of copper storage, we analyzed the localization of CUA-1.1; like the zinc transporter CDF-2, the copper transporter CUA-1.1 localizes to the expansion compartment in excess copper. These results indicate that the same intestinal lysosomes may store zinc and copper, and lysosome remodeling characterized by an increase in volume of the expansion compartment is not specific to zinc but is a more general phenomenon during metal storage in lysosomes.

## Results

### Excess zinc, copper, manganese, and cadmium induce morphological remodeling of lysosomes

Excess zinc causes morphological changes in lysosomes in intestinal cells; remodeled lysosomes are characterized by an increase in the volume of the expansion compartment. To determine if this response is specific for zinc, we analyzed the response to the physiological transition metals copper and manganese and the heavy metal cadmium. To identify a suitable concentration for each metal, we performed dose-response experiments and identified toxic levels for each metal based on larval growth rates. We then used a high concentration that was not toxic. L4 stage animals expressing CDF-2::GFP and ZIPT-2.3::mCherry were exposed to 200µM supplemental zinc [20, 32], 100 μM supplemental copper [33], 20 mM supplemental manganese, or 200 μM cadmium for 24 h on NAMM dishes containing *Escherichia coli* OP50 supplemented with LysoTracker Blue. CDF-2 is a zinc importer that localizes to the expansion compartment and the acidified compartment, whereas ZIPT-2.3 is a zinc exporter that localizes to the acidified compartment but not the expansion compartment [20]. LysoTracker also localizes to the acidified compartment but not the expansion compartment [19, 20]. Lysosomes from control worms cultured on NAMM displayed a spherical morphology; CDF-2::GFP, ZIPT-2.3::mCherry, and LysoTracker Blue were highly colocalized (Fig. 1A, B). In excess zinc, the expansion compartment appeared to significantly increase in volume (Fig. 1A, C). To quantify this morphological change, we measured the volume of the acidified compartment and the expansion compartment from 3-dimensional confocal z-stacked images. Compared to animals on control medium, animals on excess zinc displayed a significant increase in total volume of the lysosome from ∼4.3 μm^3^ to ∼18.2 μm^3^ (∼4.2X increase) (Fig. S1A, Table S1). The volume of the acidified compartment did not change significantly, whereas the volume of the expansion compartment increased significantly from ∼0.73 μm^3^ to ∼15.6 μm^3^ (∼21X increase) (Fig. S1C, E). For animals cultured in replete zinc, the expansion compartment represents ∼33% of the total volume of the lysosome—this increased significantly to ∼83% in zinc excess (Fig. S1B, D, F). These results are consistent with previous reports [19, 20].

**Figure 1:**
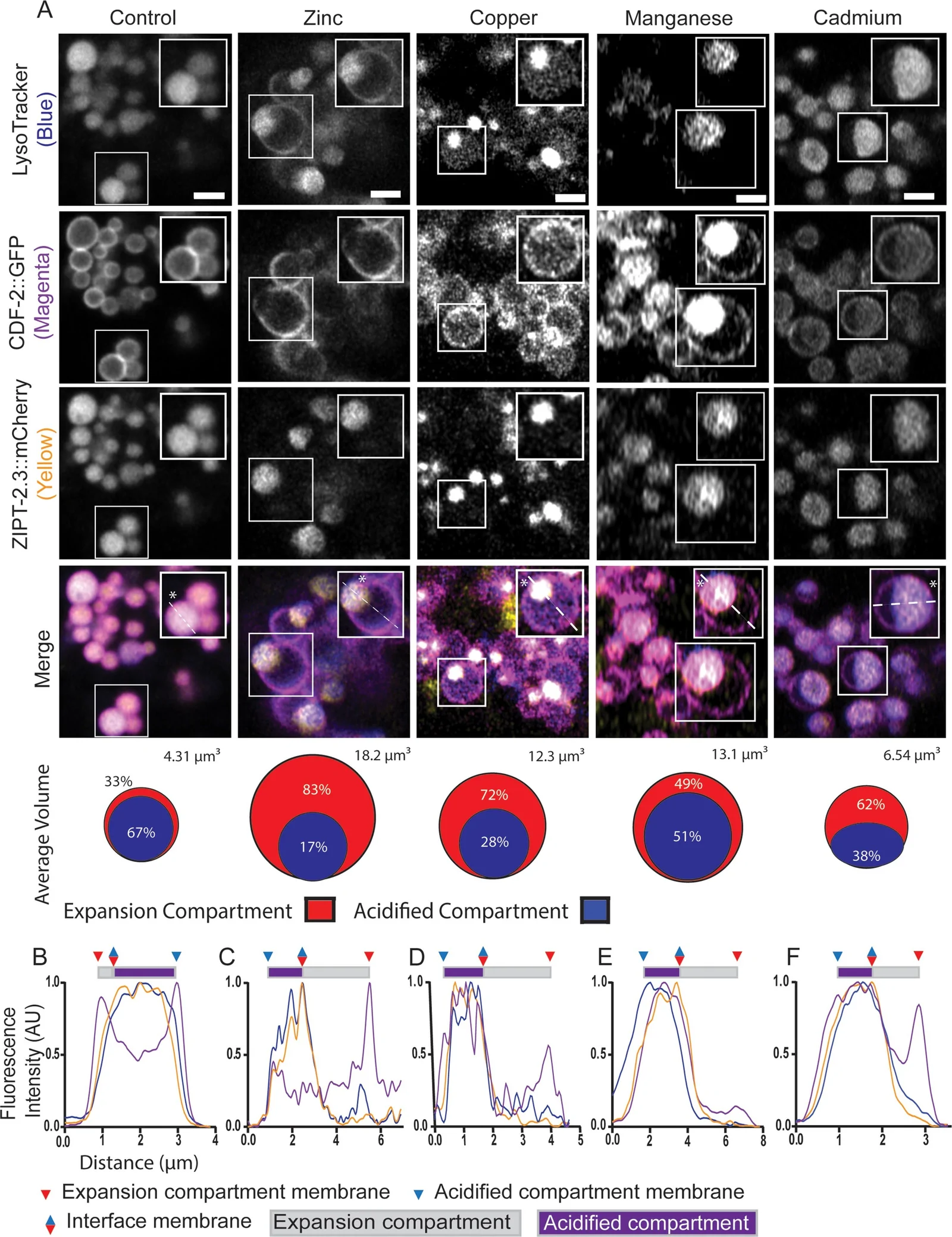
Excess zinc, copper, manganese, and cadmium induce morphological remodeling of lysosomes. (A) Transgenic L4 stage animals expressing CDF-2::GFP (magenta) and ZIPT-2.3::mCherry (yellow) were cultured for 24 h in LysoTracker Blue (blue) on either NAMM medium (control), or NAMM medium containing 200 µM supplemental zinc, 100 μM supplemental copper, 20 mM supplemental manganese, or 200 μM cadmium, and imaged by confocal super-resolution microscopy. Images show a portion of the intestine of 1-day-old adult animals. White boxes are enlarged in the upper right of each image; Scale bar = 2 µm. Dashed white lines and asterisks indicate the lysosome analyzed below. Cartoons illustrate the average total volume of a lysosome (μm^3^) and the % represented by the acidified (blue) and expansion (red) compartments (Fig. S1, Table S1). (B-F) Line scans of individual lysosomes in control (B), excess zinc (C), excess copper (D), excess manganese (E), and cadmium (F). Fluorescence intensity along the path of the line was measured beginning at the white asterisk. CDF::GFP, ZIPT-2.3:mCherry, and LysoTracker, are displayed as magenta, yellow, and blue, respectively. For each color line, the highest value of fluorescence intensity is set to 1.0 in arbitrary units (AU), and the lowest fluorescence intensity is set to 0. All other values are normalized. Triangles indicate positions of membranes, and rectangles indicate lysosomal compartments. Representative images are shown from ≥ 2 experiments per condition with > 3 worms per experiment.

In excess copper, the expansion compartment significantly increased in volume (Fig. 1A, D). Compared to animals on control medium, animals on excess copper had an increase in total volume of the lysosome from ∼4.3 μm^3^ to ∼12.3 μm^3^ (∼3X increase) (Fig. S1A, Table S1). The volume of the acidified compartment did not change significantly, whereas the volume of the expansion increased significantly from ∼0.73 μm^3^ to ∼10.1 μm^3^ (∼14X increase) (Fig. S1C, E). In control animals, the expansion compartment represents ∼33% of the total volume of the lysosome—this increased significantly to ∼72% in copper excess (Fig. S1B, D, F, Table S1). These results suggest that excess copper may be stored in lysosomes.

In excess manganese, the expansion compartment significantly increased in volume (Fig. 1A, E). Compared to animals on control medium, animals on excess manganese have an increased total volume of lysosomes from ∼4.3 μm^3^ to ∼13.2 μm^3^ (Fig. S1A, Table S1). The volume of the acidified compartment did not change significantly, whereas the volume of the expansion increased significantly from ∼0.73 μm^3^ to ∼7.7 μm^3^ (∼11X increase) (Fig. S1C, E, Table S1). In control animals, the expansion compartment represents ∼33% of the total volume of the lysosome—this increased to ∼49% in manganese excess; this effect was not statistically significant with this sample size (Fig. S1B, D, F, Table S1).

Cadmium is not added to standard medium. Exposure to 200 μM cadmium appeared to cause lysosome remodeling suggestive of an increase in the volume of the expansion compartment (Fig. 1A, F). The expansion compartments were typically shaped like a hemisphere. Compared to animals on control medium, animals on cadmium displayed an increase in total volume of the lysosome from ∼4.3 μm^3^ to ∼6.5 μm^3^ (∼1.5X increase); however, this trend was not statistically significant with this sample size (Fig. S1A, Table S1). The volume of the acidified compartment did not change significantly, whereas the volume of the expansion increased from ∼0.73 μm^3^ to ∼4.2 μm^3^ (∼5.8X increase); however, this trend was not statistically significant with this sample size (Fig. S1C, E). In control animals, the expansion compartment represents ∼33% of the total volume of the lysosome—this increased significantly to ∼62% in cadmium exposed animals (Fig. S1B, D, F). These results suggest that exposure to cadmium triggers lysosome remodeling.

### Lysosomes contribute to detoxification of excess zinc, copper, and manganese

If lysosomes are a storage site for a metal, then we predict that reducing the number of lysosomes might have two effects: (i) Animals will become hypersensitive to metal excess, because lysosomes are not available for storage. (ii) Animals will become hypersensitive to metal deficiency, because lysosomes are not available as a source of stored metal. To test these predictions, we analyzed *glo-1(zu391)* mutant animals that are characterized by a reduced number of intestinal lysosomes [34]. We measured growth of larvae after 24 h starting at the L4 stage, because the length of individual animals can be accurately and precisely quantified and serves as a good indicator of overall health. Wild-type and *glo-1(zu391)* animals were cultured in a variety of concentrations of supplemental metal and metal chelators to test metal excess and deficiency, respectively. The *glo-1(zu391)* loss-of-function mutation was isolated in a forward mutagenesis screen, and the strain was then backcrossed multiple times to eliminate background mutations [34]. However, this procedure does not eliminate the possibility of residual background mutations, and our results analyzing this mutant strain do not exclude the possibility that background mutations remain in this strain that can influence the phenotype. In the absence of supplements (0 μM), wild type and *glo-1(zu391)* mutants displayed similar lengths, indicating that the *glo-1(zu391)* mutant strain does not have a growth defect in standard conditions (Fig. 2). Thus, if there are background mutations in this strain, they do not cause a measurable growth defect in standard conditions.

**Figure 2:**
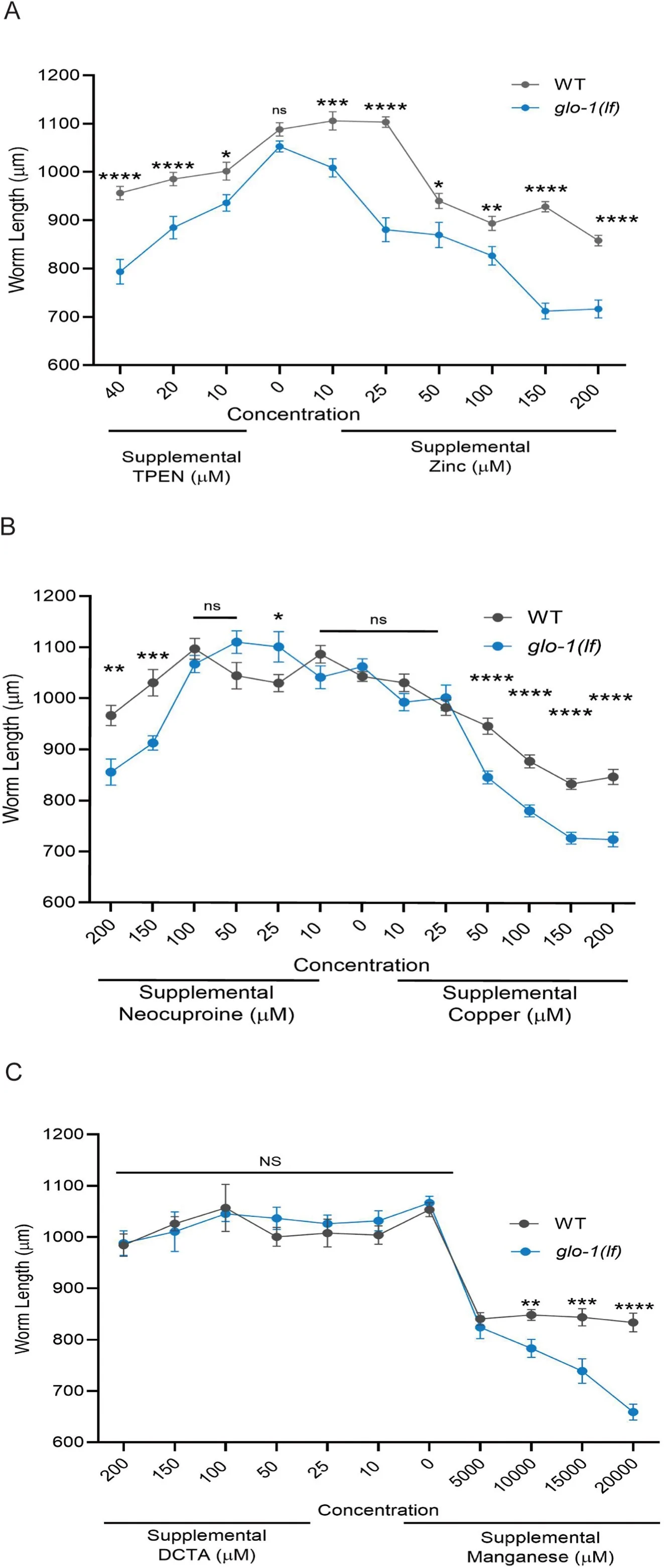
*glo-1(lf)* mutants with reduced numbers of lysosomes are hypersensitive to metal excess and deficiency. Wild-type or *glo-1(zu391)* loss-of-function animals were synchronized at the L4 stage and cultured for 24 h on standard NAMM medium or NAMM medium containing supplemental metal or chelator. The length of individual worms was measured using image analysis in FIJI. (A). NAMM medium containing the zinc chelator TPEN or supplemental zinc. Values represent the average length ± SEM. *N* = 25−43 animals (wild type) and 30−46 animals (*glo-1(lf))*. (B) NAMM medium containing the copper chelator Neocuproine or supplemental copper. *N* = 27−44 animals (wild type) and 26−44 animals (*glo-1(lf))*. (C) NAMM medium containing the manganese chelator DCTA or supplemental manganese. *N* = 18−45 animals (wild type) and 23−47 animals (*glo-1(lf))*. Two-way Anova: NS, not significant, *P ≤ .05, **P ≤ .01, ***P ≤ .001, ****P ≤ .0001.

With low concentrations of supplemental zinc (10 and 25 μM), wild-type worms displayed normal growth, whereas *glo-1(zu391)* mutants displayed significantly less growth (Fig. 2A). In higher concentrations of supplemental zinc (50−200 μM), wild-type worms displayed reduced growth, whereas *glo-1(zu391)* mutants displayed hypersensitivity (significantly less growth than wild type). Thus, *glo-1(zu391)* animals are hypersensitive to both mild and extreme zinc excess. To create zinc deficient conditions, we utilized the zinc chelator N, N, N', N'-tetrakis(2-pyridylmethyl)ethane-1,2-diamine (TPEN) [35]. Wild-type animals cultured on 10, 20, or 40 μM TPEN displayed reduced growth, indicating sensitivity to these levels of zinc deficiency, whereas *glo-1(zu391)* mutants displayed hypersensitivity (significantly less growth than wild type) (Fig. 2A). We interpret these results as indicating that lysosomes promote resistance to both zinc excess and deficiency, consistent with the model that lysosomes are a storage site for zinc. These findings are consistent with previous results. Roh et al. [19] showed that loss-of-function mutations in LRO biogenesis genes *glo-1, pgp-2*, and *glo-3* reduced intestinal zinc levels and caused hypersensitivity to growth in excess zinc; because three different genes were analyzed, this finding is unlikely to be caused by background mutations. Furthermore, Mendoza et al. [20] observed that *glo-1(zu391)* mutants were hypersensitive to zinc excess and deficient conditions.

With low concentrations of supplemental copper (10 and 25 μM), wild-type worms and *glo-1(zu391)* mutants displayed similar growth (Fig. 2B). In higher concentrations of supplemental copper (50−200 μM), wild-type worms displayed reduced growth, whereas *glo-1(zu391)* mutants displayed hypersensitivity (significantly less growth than wild type) (Fig. 2B). Thus, *glo-1(zu391)* animals are hypersensitive to extreme copper excess. To create copper deficient conditions, we utilized the copper chelator Neocuproine [36]. Wild-type animals and *glo-1(zu391)* mutants cultured on 10−100 μM Neocuproine displayed growth that was similar to replete copper. When cultured with 150 or 200 μM Neocuproine, wild-type animals displayed reduced growth, indicating sensitivity to these levels of copper deficiency, whereas *glo-1(zu391)* mutants displayed hypersensitivity (significantly less growth than wild type) (Fig. 2B). Thus, lysosomes promote resistance to both copper excess and deficiency, consistent with the model that lysosomes are a storage site for copper.

With low concentrations of supplemental manganese (5000 μM), wild-type worms and *glo-1(zu391)* mutants displayed a similar reduction in growth (Fig. 2C). In higher concentrations of supplemental manganese (10 000−20 000 μM), wild-type worms displayed reduced growth, whereas *glo-1(zu391)* mutants displayed hypersensitivity (significantly less growth than wild type). Thus, *glo-1(lf)* animals are hypersensitive to extreme manganese excess. These results indicate that lysosomes promote resistance to manganese excess, consistent with the model that lysosomes may be a storage site for manganese. The concentration of supplemental manganese necessary to induce growth defects was substantially higher than the concentrations of supplemental zinc and copper that induced growth defects. This is consistent with the previously established LC_50_ values in *C. elegans* for manganese (7142.2 mg/L [37]), zinc (347 mg/L), and copper (170 mg/L) [38]. In principle, the amount of supplemental metal necessary to cause a growth defect depends on two factors: (i) The inherent toxicity of the metal when it is in excess and (ii) the ability of the organism to maintain homeostasis of the metal. One possibility is that zinc and copper are inherently more toxic than manganese, so that lower concentrations of these metals induce growth defects. Alternatively, manganese homeostasis might be more effective than zinc or copper homeostasis; thus, even though we used larger amounts of supplemental manganese, an effective homeostasis system might result in a low level of manganese excess in the animal.

To attempt to create manganese deficient conditions, we utilized the manganese chelator 1,2-Diaminocyclohexanetetraacetic acid monohydrate (DCTA) [39, 40]. Wild type and *glo-1(zu391)* mutant animals cultured on 10−200 μM DCTA did not display reduced growth (Fig. 2C); these results do not test the model, because the highest levels of chelator did not cause a phenotype. One possible interpretation is that both strains can tolerate this level of manganese deficiency without displaying a measurable growth defect. Alternatively, the chelator may not reduce the concentration of manganese in worms under these conditions. Future experiments using higher concentrations of chelator or investigating other phenotypes such as the function of Mn-dependent enzymes might distinguish between these alternatives.

### Measurements of metals by XFM in isolated lysosomes

To investigate the hypothesis that lysosomes in intestinal cells store multiple metals, we developed a technique to quantify the metal content of isolated lysosomes. Previous studies in *C. elegans* have used the zinc dye FluoZin3 and the copper (I) dye CF4 to stain intact animals, which indicates that lysosomes accumulate zinc and copper, respectively [18–20, 31, 41]. However, these dyes have limitations, and a manganese dye has not been employed. To quantitatively evaluate the metal content of lysosomes, we utilized the Bionanoprobe (BNP) at Argonne National Lab to conduct XFM [42]. The BNP permits studies from biological samples at sub-100 nm resolution, within a range of 4.5−35 KeV [43] making it best suited to visualize metal content in lysosomes. XFM uses high-energy X-rays to ionize inner electrons of atoms; in this unstable state, electrons from the outer shells drop down to fill those electron vacancies, thereby emitting energy in the form of X-ray fluorescence photons. Each element can be quantified in the same experiment based on its unique output. XFM has previously been used to analyze intact *C. elegans* [43, 44]; to focus the analysis on lysosomes, we developed a method to dissect worms and expel intestinal lysosomes onto a grid suitable for XFM analysis. Transgenic animals were cultured in either control or excess zinc medium for 24 hours, and lysosomes were isolated by dissecting out the intestine, allowing the lysosomes to flow into the buffer, and pipetting the lysosomes onto a silicon nitride window for XFM analysis. To analyze the images, we measured the concentration of nine different elements inside the lysosome, subtracted background, and calculated the absolute number of each atom in the lysosome. The approach we developed has differences from previous *C. elegans* XFM protocols. For example, in Ganio et al. (2016) [43], whole hydrated nematode samples were prepared for XFM, and in Hare et al. [44], nematode samples were flash frozen and lyophilized onto silicon nitride windows. By contrast, we air-dried isolated lysosomes onto silicon nitride windows, similar to the strategy described by Mendoza et al. [45] for analysis of the germline. By isolating lysosomes from the intestine, we eliminate potential elemental counts from other regions of the intestine, the germline, or the cuticle.

Each of the nine elements displayed a characteristic pattern of distribution within the spherical lysosome (Fig. 3A−I, K−N). Phosphorus, calcium, and iron appeared at the perimeter of the lysosome, whereas sulfur, chlorine, potassium, manganese, copper, and zinc appeared to be distributed throughout the lumen of the lysosome. We calculated the average number of atoms in each lysosome; there were two general classes of abundance (Fig. 3J). Phosphorus (1.6*10^9^ atoms per lysosome), sulfur (1.1*10^9^ atoms per lysosome), chlorine (6.0*10^8^ atoms per lysosome), potassium (1.5*10^9^ atoms per lysosome), and calcium (5.7*10^8^ atoms per lysosome) were relatively abundant, in the range of 10^9^ atoms per lysosome. By contrast, manganese (3.8*10^6^ atoms per lysosome), iron (4.5*10^6^ atoms per lysosome), copper (8.6*10^5^ atoms per lysosome), and zinc (7.8*10^6^ atoms per lysosome) were relatively less abundant, in the range of 10^6^−10^7^ atoms per lysosome.

**Figure 3:**
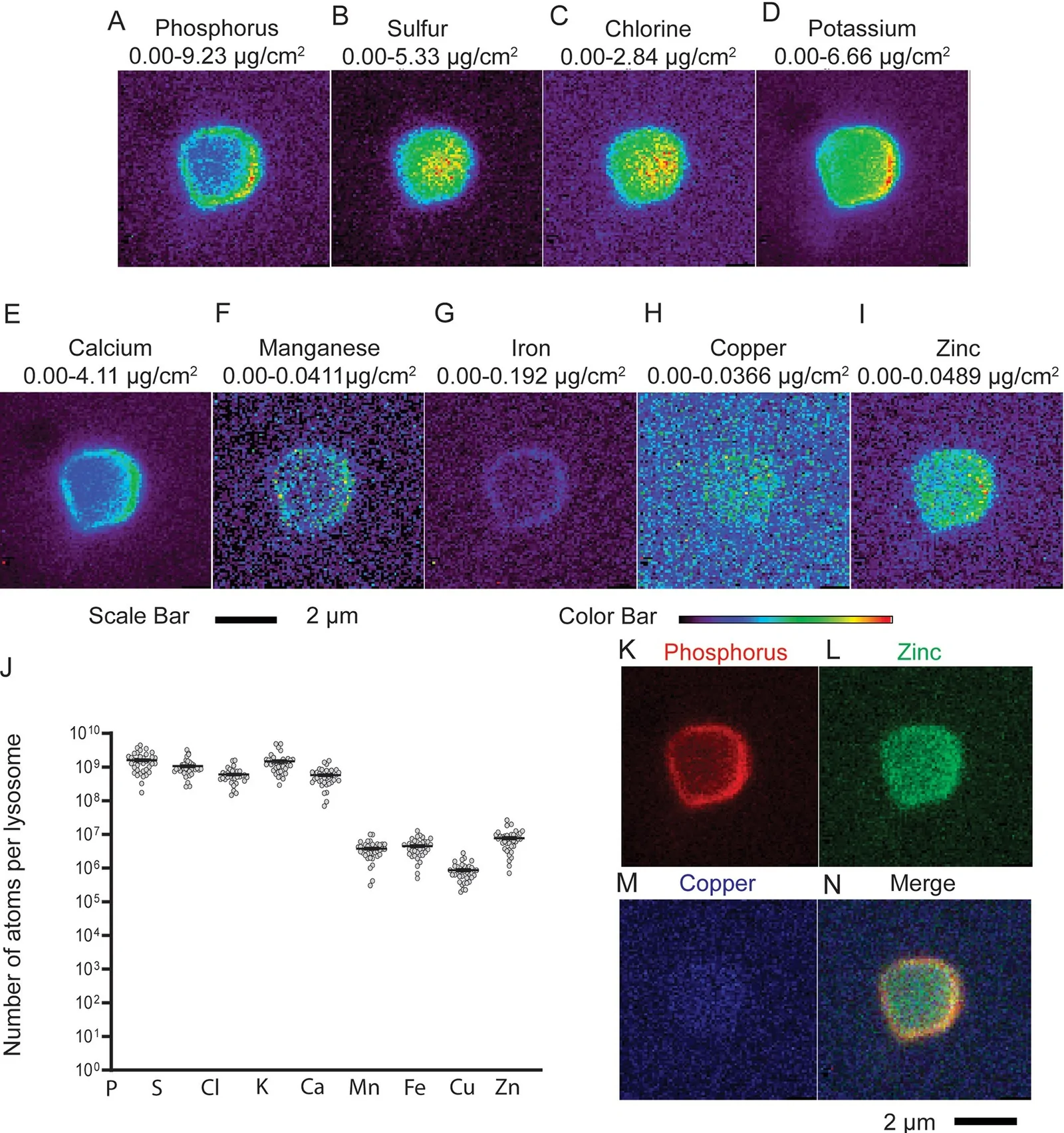
XFM analysis of individual lysosomes in standard medium. (A−I) Transgenic animals expressing CDF::GFP and ZIPT-2.3::mCherry were cultured on standard NAMM dishes. Intestinal lysosomes were isolated by dissection one day after the L4 stage, and XFM images were acquired with a 0.08 μm step size and a dwell time of 500 ms. Panels display XFM images from one representative lysosome, illustrating the distribution and intensity of phosphorus, sulfur, chlorine, potassium, calcium, manganese, iron, copper, and zinc. Color bar represents the spectrum of intensity; red is highest, and black is lowest. The extreme high and low intensity for each element is shown as the concentration range (μg/cm^2^). Scale bar = 2 μm. (J) Each data point represents one lysosome; bars and whiskers represent the mean ± SEM. (*N* = 37 lysosomes from 3 worms from one experiment). (K−N) False colored XFM images of phosphorous (red), zinc (green), and copper (blue); a merge displays overlaps in localization of these elements. The images are the same data as (A), (H), and (I). Scale bar = 2 μm.

To directly test the model that zinc is stored in lysosomes in response to dietary excess, we performed a similar analysis with 200 μM supplemental zinc in the medium (Fig. 4). We quantified 60 individual lysosomes from multiple animals in zinc excess medium and compared the results to 37 animals cultured on control medium. Expansion compartments cannot be seen in these images as the lysosomes were flattened for this analysis. Among the five abundant elements, the number of atoms per lysosome for sulfur, chlorine, and calcium was not significantly different from control, whereas phosphorus and potassium were significantly lower than control (Fig. S2A−E). Among the four less abundant transition metal elements, the number of atoms per lysosome for iron and copper was not significantly different from control, whereas manganese was significantly lower than control (Fig. S2F−H). For zinc, the number of atoms per lysosome increased significantly (13.8-fold increase), consistent with the model that zinc is stored in lysosomes in response to dietary excess (Figs. 4K and S2I).

**Figure 4:**
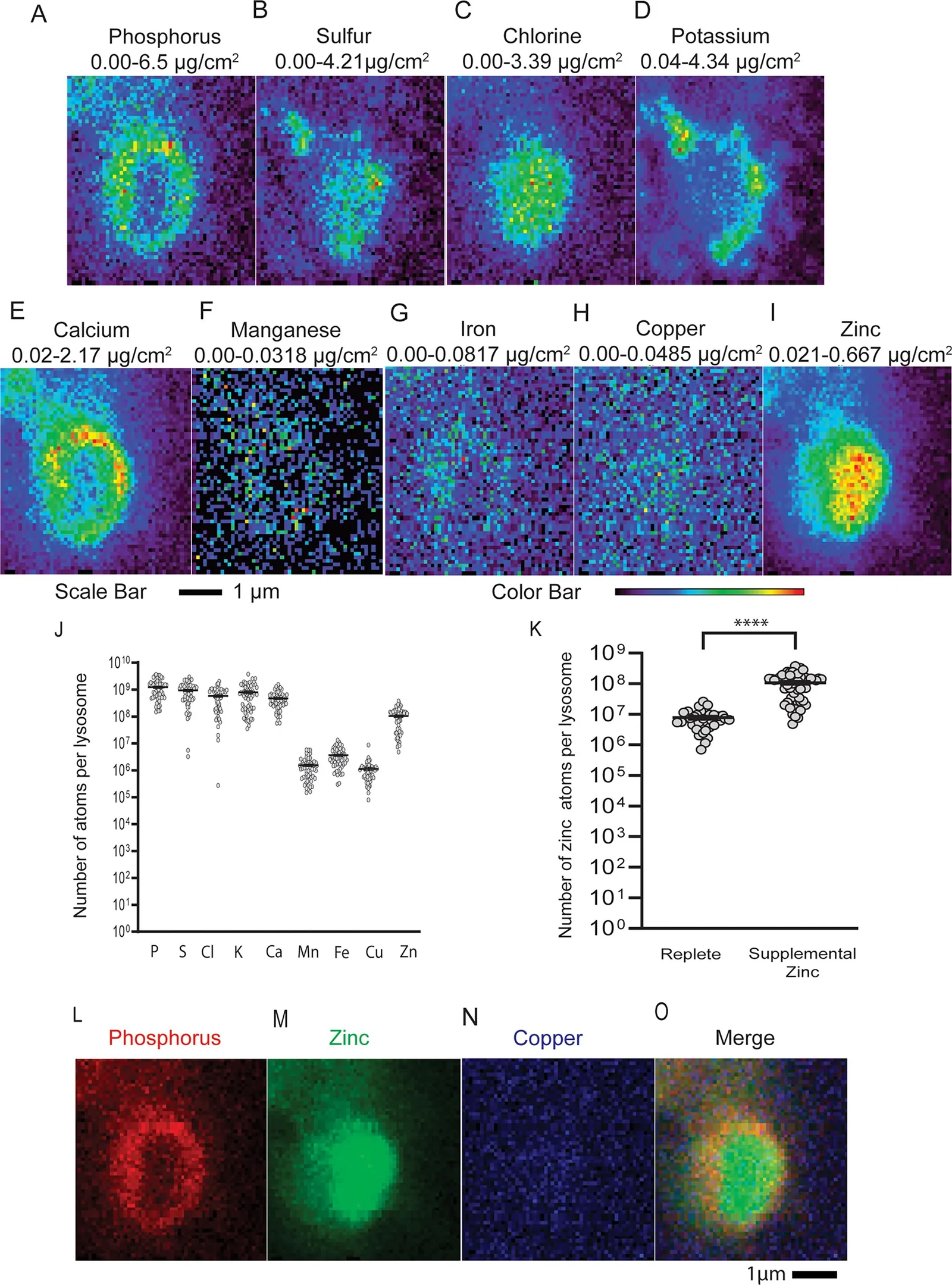
XFM analysis of individual lysosomes in excess zinc medium. (A−I) L4 stage transgenic worms expressing CDF::GFP and ZIPT-2.3::mCherry were transferred to NAMM dishes containing 200 μM supplemental zinc and cultured for 24 h. Intestinal lysosomes were isolated by dissection from young adult animals, and XFM images were acquired with a 0.08 μm step size and a dwell time of 500 ms. Panels display XFM images from one representative lysosome, illustrating the distribution and intensity of phosphorus, sulfur, chlorine, potassium, calcium, manganese, iron, copper, and zinc. Color bar represents the spectrum of intensity; red is highest, and black is lowest. The extreme high and low intensity for each element is shown as the concentration range (μg/cm^2^). Scale bar = 1 μm. (J) Each data point represents one lysosome; bars and whiskers represent the mean ± SEM. (*N* = 60 lysosomes from 3 worms from one experiment). (K) Comparison of the number of zinc atoms per lysosome from animals cultured in standard NAMM (replete) or 200 μM supplemental zinc (excess) was performed by Mann Whitney t-test. ****P ≤ .0001. (L−O) False colored XFM images of phosphorous (red), zinc (green), and copper (blue); a merge displays overlaps in localization of these elements. The images are the same data as (A), (H), and (I). Scale bar = 1 μm.

### The copper transporter CUA-1.1 localizes to both the acidified and expansion compartments

The *C. elegans cua-1.1* gene encodes a member of the ABC transporter family that functions as a copper transporter [31]. CUA-1.1 protein localizes to the basolateral membrane of intestinal cells in replete copper, indicating it transports copper from the cytosol of intestinal cells into the pseudocoelomic space. However, in excess copper conditions, CUA-1.1 relocalizes to the membrane of lysosomes, indicating it transports copper from the cytosol of intestinal cells into the lumen of the lysosome to sequester excess copper. These localization studies were performed using confocal microscopy, and the status of the expansion compartment was not reported [31]. We hypothesized that excess copper would cause the expansion compartment to increase in volume as a result of delivering CUA-1.1 to the lysosome. To determine the localization of CUA-1.1 in the lysosome, we analyzed a transgenic strain that expresses CUA-1.1 fused to GFP under the control of the *vha-6* promoter (P*_vha-6_*::CUA-1.1::GFP::unc-54 3′ UTR) [31]. In addition, we monitored copper (I) localization using the copper dye CF4 [46, 47]. To validate the CF4 copper dye, we cultured wild-type animals with supplemental copper or the copper (I) chelator BCS. In copper (II) excess, lysosomes in intestinal cells displayed bright fluorescence with CF4; by contrast, in copper deficiency, the CF4 fluorescence was dim (Fig. S3A, B). Quantitive analysis revealed a significant difference between these two conditions, indicating that the CF4 dye is copper (I) responsive in *C. elegans*, consistent with previous studies (Fig. S3C) [31].

To resolve the acidified and expansion compartments, we performed super-resolution confocal fluorescence microscopy. We analyzed two separate transgenic worm strains; one expresses CDF-2 fused to GFP under the control of the *cdf-2* promoter; CDF-2 localizes to both the expansion compartment and the acidified compartment [20]. The other strain expresses CUA-1.1::GFP. Each strain was cultured on NAMM dishes with *E. coli* OP50 supplemented with LysoTracker blue and the copper dye CF4 (Fig. 5). In replete copper medium, lysosomes appear spherical with a very small expansion compartment that represents ∼13% of the volume (Fig. S4, Table S2); CDF-2::GFP displayed an extensive overlap with LysoTracker and CF4 (Fig. 5A). By contrast, CUA-1.1::GFP displayed weak staining of lysosomes, indicating that CUA-1.1 must be primarily localized to the basolateral membrane; the spherical lysosomes displayed a very small expansion compartment that represents ∼7% on average of the volume. (Fig. S4, Table S2); CUA-1.1::GFP displayed an overlap with LysoTracker and CF4 (Fig. 5B). We note that the % of the total lysosome volume represented by the expansion compartment was lower in these strains (∼13% and ∼7%) compared to the strain containing CDF-2::GFP and ZIPT-2.3::mCherry analyzed in Fig. 1 (∼33%). This variability might reflect a true difference between the strains or variability in measurements performed independently in different strains.

**Figure 5:**
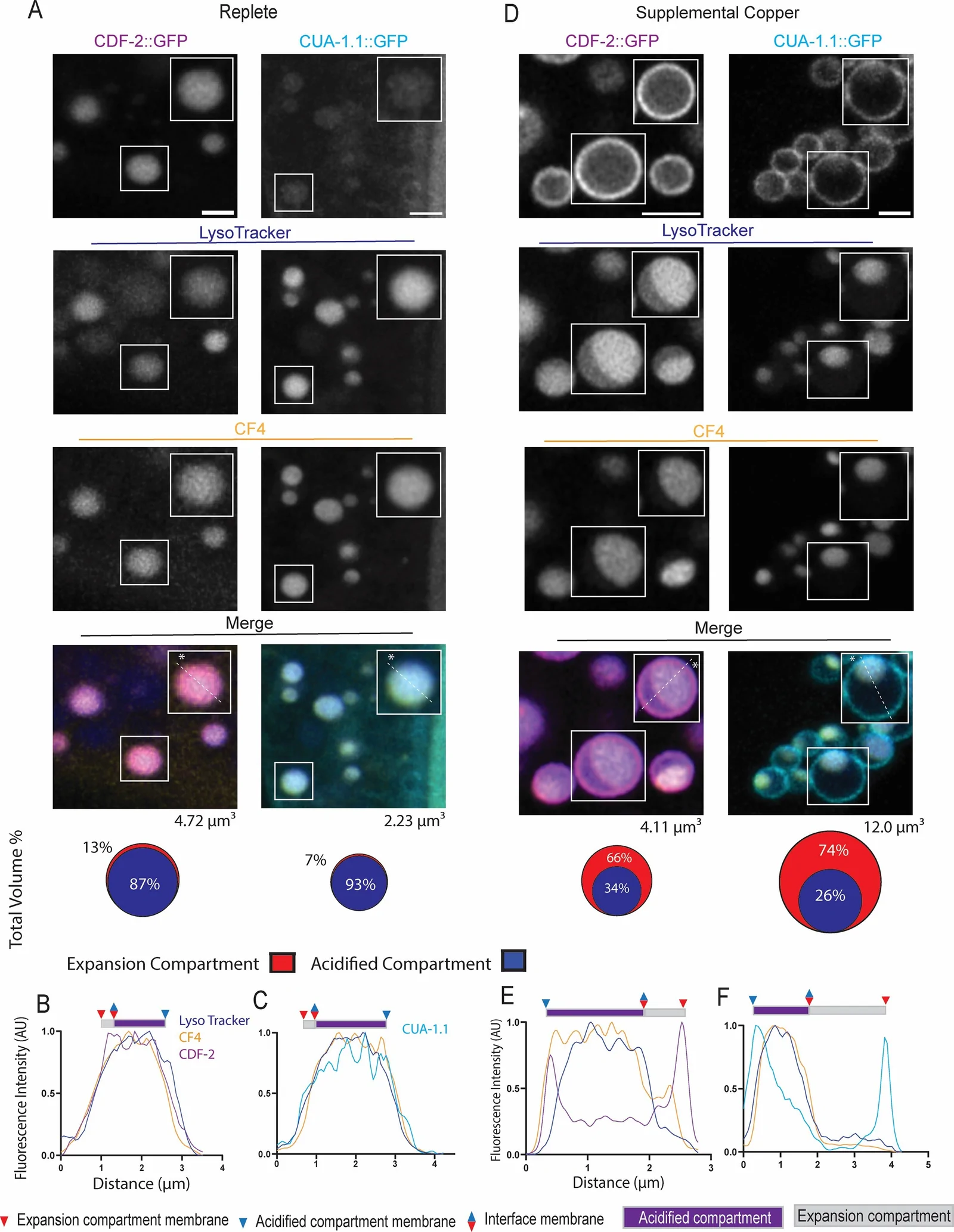
The copper transporter CUA-1.1 localizes to the expansion compartment and acidified compartment membranes in excess copper. (A) Transgenic L4 stage animals expressing CDF-2::GFP (magenta) or CUA-1.1::GFP (cyan) were cultured for 24 h in LysoTracker Blue (blue) and the copper dye CF4 (yellow) on either NAMM medium (replete), or NAMM medium containing 100 μM supplemental copper, and imaged by confocal super-resolution microscopy. Images show a portion of the intestine of 1-day old adult animals. White boxes are enlarged in the upper right of each image. Scale bar = 2 µm. Dashed white lines and asterisks indicate the lysosome analyzed below. Cartoons illustrate the average total volume of a lysosome (μm^3^) and the % represented by the acidified (blue) and expansion (red) compartments (Fig. S4, Table S2). (B-F) Line scans of individual lysosomes in replete (B, C) or excess copper (E, F). Fluorescence intensity along the path of the line was measured beginning at the white asterisk. CDF::GFP, CUA-1.1::GFP, LysoTracker, and CF4 are displayed as magenta, cyan, blue, and yellow, respectively. For each color line, the highest value of fluorescence intensity is set to 1.0 in AU, and the lowest fluorescence intensity is set to 0. All other values are normalized. Triangles indicate positions of membranes, and rectangles indicate lysosomal compartments. Representative images of *N* = 3 worms per condition.

In excess copper, lysosomes displayed a significant increase in the volume of the expansion compartment to ∼66% of the total volume in the CDF-2::GFP strain and ∼74% of the total volume in the CUA-1.1::GFP strain (Fig. S4, Table S2). CDF-2::GFP localized to the acidified compartment and the expansion compartment. By contrast, LysoTracker and CF4 localized to the acidified compartment but not the expansion compartment (Fig. 5D, E). In excess copper, CUA-1.1::GFP displayed a similar pattern to CDF-2::GFP; CUA-1.1::GFP localized to acidified compartment and the expansion compartment, whereas CF4 and LysoTracker localized to the acidified compartment but not the expansion compartment (Fig. 5D, F).

### CUA-1.1 and CDF-2 localize to the same lysosomes in intestinal cells

*Caenorhabditis elegans* intestinal cells contain many lysosomes. One possible model is that zinc storing lysosomes are separate from copper storing lysosomes; alternatively, the same lysosomes may store both zinc and copper. To distinguish these models, we generated a transgenic strain that expresses both CDF-2 fused to mCherry under the control of the *cdf-2* promoter and CUA-1.1::GFP described above. In copper replete medium, confocal super-resolution microscopy revealed that CUA-1.1::GFP localized primarily to the basolateral membrane and weakly to the lysosomal membrane; by contrast, CDF-2::mCherry localized specifically to lysosomes (Fig. 6A). The same lysosomes that were strongly fluorescent for CDF-2::mCherry were also weakly fluorescent for CUA-1.1::GFP, demonstrating that both transporters reside on the same lysosomes in replete medium (Fig. 6B, C). To determine the effect of excess copper, we cultured these animals in supplemental copper for 24 hours with *E. coli* OP50 containing LysoTracker. Confocal super-resolution microscopy revealed that lysosomes were remodeled; the % of the lysosome volume occupied by the expansion compartment increased from ∼30% in copper replete medium to ∼67% in excess copper medium (Fig. S5, Table S3). Importantly, CDF-2::mCherry and CUA-1.1::GFP co-localized to both the acidified and expansion compartments of the same lysosomes (Fig. 6B, D). In these conditions, the two transporters displayed a highly overlapping pattern. These results indicate that the same lysosomes contain zinc and copper transporters and likely store both metals.

**Figure 6:**
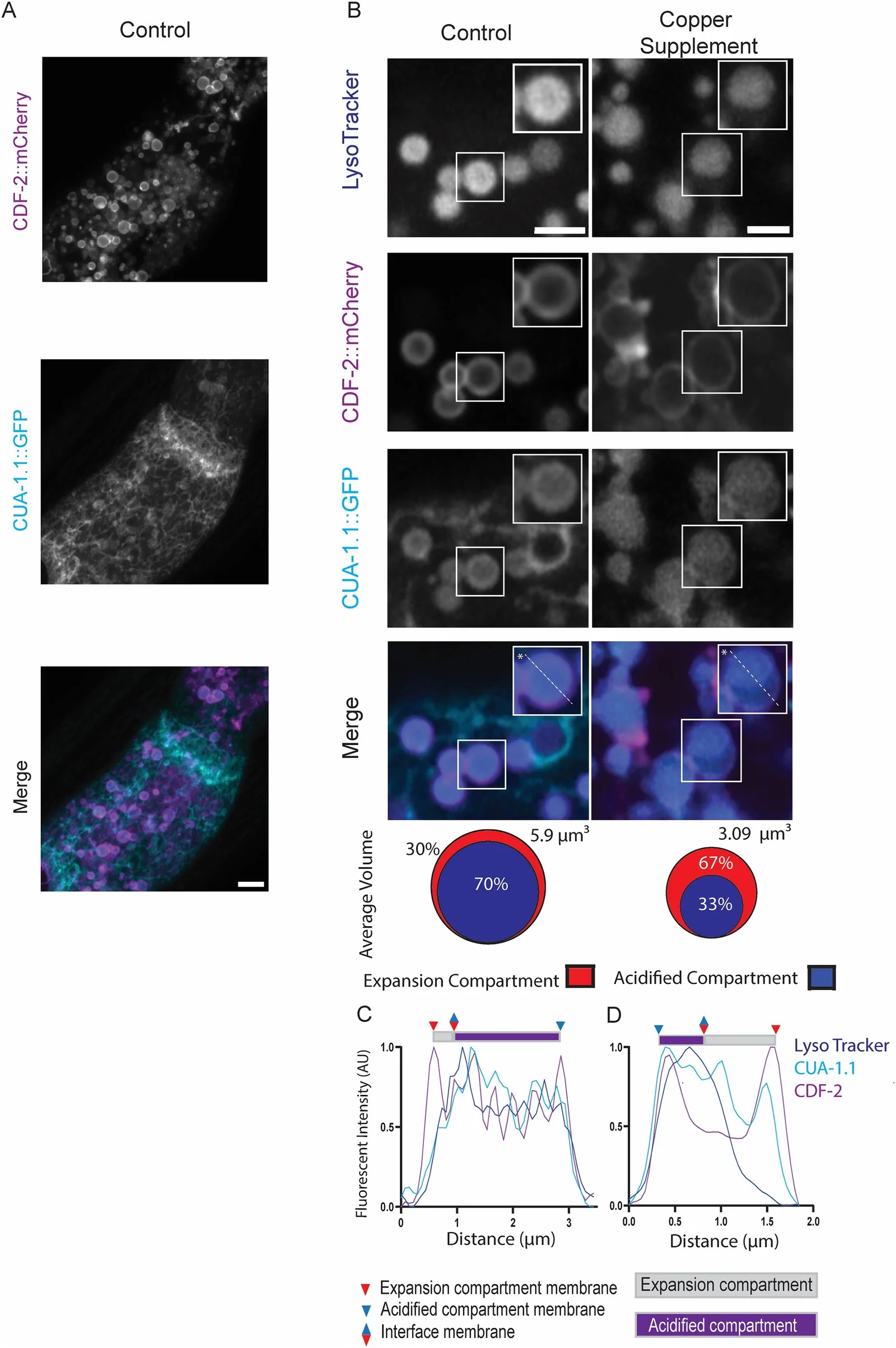
CDF-2 and CUA-1.1 localize to the same lysosomes. (A) Transgenic L4 stage animals expressing CDF-2::mCherry (magenta) and CUA-1.1::GFP (cyan) were cultured for 24 h on NAMM medium (control) and imaged by confocal super-resolution microscopy. Images show a portion of the intestine of 1-day old young adult animals. Scale bar = 2 µm. (B) The same strain was cultured for 24 h in LysoTracker Blue (blue) on either NAMM medium (control), or NAMM medium containing 100 μM supplemental copper (copper supplement), and imaged by confocal super-resolution microscopy. Images show a portion of the intestine of 1-day old young adult animals. White boxes are enlarged in the upper right of each image; Scale bar = 2 µm. Dashed white lines and asterisks indicate the lysosome analyzed below. Cartoons illustrate the average total volume of a lysosome (μm^3^) and the % represented by the acidified (blue) and expansion (red) compartments (Fig. S5, Table S3). (C, D) Line scans of individual lysosomes in replete (C) or excess copper (D). Fluorescence intensity along the path of the line was measured beginning at the white asterisk. CDF::mCherry, CUA-1.1::GFP, and LysoTracker are displayed as magenta, cyan, and blue, respectively. For each color line, the highest value of fluorescence intensity is set to 1.0 in AU, and the lowest fluorescence intensity is set to 0. All other values are normalized. Triangles indicate positions of membranes, and rectangles indicate lysosomal compartments. Representative images of *N* = 3 worms per condition.

## Discussion

Transition metals are essential for all organisms because of their versatile chemical properties. However, this necessity for metals has a downside—excess metals are toxic and must be regulated carefully. In the case of iron, excess metal is stored with the protein ferritin [48, 49]. In the case of other metals, metallothionein proteins in the cytosol are used to sequester excess. Lysosomes are emerging as an important site of excess metal sequestration; these lysosomes also provide a source of metal in response to deficiency. Here we used *C. elegans* intestinal cells to probe the generality of excess metal storage in lysosomes by analyzing zinc, copper, manganese, and cadmium. In *C. elegans*, analysis with the zinc dye FluoZin-3 indicated that intestinal lysosomes store excess zinc [19]. Confocal super-resolution microscopy revealed that lysosomes contain two compartments, a LysoTracker positive acidified compartment, and a LysoTracker negative expansion compartment [20]. The zinc transporters CDF-2 and ZIPT-2.3 localize to the acidified compartment, whereas CDF-2 but not ZIPT-2.3 localizes to the expansion compartment. In excess zinc, CDF-2 mRNA and protein are upregulated, and CDF-2 positive vesicles fuse to the expansion compartment, resulting in an increase in volume and the amount of CDF-2 protein. Based on these observations, Mendoza et al. [20] proposed that the increase in the volume of the expansion compartment is caused by the fusion of vesicles delivering CDF-2 protein. To test the hypothesis that the increase in the expansion compartment volume is a general mechanism to deliver metal transporters and increase the capacity to store metals, we investigated additional metals. In addition, we developed a technique to quantify metals in isolated lysosomes using XFM. In replete zinc medium, we measured 7.8*10^6^ zinc atoms per lysosome, which appears to be distributed throughout the lumen of the lysosomes. In excess zinc medium, the amount increased dramatically to 1.0*10^8^ zinc atoms per lysosome. This result is consistent with the conclusion based on the dye FluoZin-3 and formally demonstrates that lysosomes are an important site of zinc storage during excess.

The observation that labile zinc (visualized by FluoZin-3) was sometimes absent and sometimes present in the expansion compartment may suggest that labile zinc was absent in the expansion compartment in one population of lysosomes, despite CDF-2 localization suggested that the zinc dye was unable to access bound zinc in the expansion compartment [19, 20]. In instances where it was present, it is possible that the expansion compartment in another population was actively mobilizing zinc. XFM revealed that total zinc was detected throughout the entire lysosome, supporting the idea that CDF-2 could be importing zinc into the expansion compartment as well as the acidified compartment. Developing an XFM procedure to isolate lysosomes is a novel approach and is necessary to understand how lysosomes acquire zinc and uncover the elements that are present within them. Previous XFM analyses in worms were performed on whole adults and isolated embryos [44, 45,50–53]. By contrast, here we demonstrate the ability to isolate individual lysosomes from intestines. This novel innovation opens opportunities for refining metal detection in organelles and correlating zinc content with compartments.

The heavy metal cadmium accumulates in animals [54, 55]. However, because cadmium does not have a physiological function in animals, this accumulation is not an evolved response to store excess metal for future use. Earley et al. [25] reported that cadmium hijacks the high zinc response by binding and activating HIZR-1, which results in the upregulation of CDF-2. Here we showed that 200 μM cadmium causes lysosome remodeling characterized by an increase in the volume of the expansion compartment. We interpret this response as an upregulation of CDF-2 transcription, translation, and delivery by vesicles to the expansion compartment. It is not known if the zinc transporter CDF-2 can also transport cadmium. Cadmium-specific dyes have not been reported, so it is unclear where cadmium accumulates in *C. elegans*.

The physiological metal manganese has been studied in *C. elegans* [23, 55]. Although manganese specific dyes have been reported [56], they are not readily available, and the localization of manganese in *C. elegans* is not well established. Here we show that excess manganese causes lysosome remodeling characterized by an increase in the volume of the expansion compartment. We speculate that this reflects the delivery of vesicles containing a manganese transporter to the expansion compartment of the lysosome to increase the capacity for manganese storage. However, this manganese transporter has not been identified. Consistent with this model, XFM revealed ∼1.5*10^6^ and 3.5*10^6^ manganese atoms per lysosome in replete and excess zinc medium, respectively. The level of manganese detected in the lysosomes was similar to the level of copper and zinc detected in the lysosomes. These experiments were performed in replete manganese. Our model does not predict the level of manganese in the lysosome under replete conditions—these conditions might have little excess manganese and thus little need for manganese storage in the lysosome. Our model predicts that supplemental manganese will cause higher levels of manganese in the lysosome; future XFM experiments could rigorously test this prediction. To evaluate the physiological importance of lysosomes for excess manganese sequestration, we analyzed *glo-1(zu391)* mutants that have a reduced number of lysosomes. These mutants were hypersensitive to excess manganese, suggesting that lysosomes store and sequester manganese. Together, these results indicate that lysosomes in intestinal cells of *C. elegans* play an important role in manganese storage. Important future experiments to test this hypothesis include the identification of the transporters that store manganese in lysosomes during excess and release manganese from lysosomes during deficiency.

Copper is an essential metal; Chun et al. [31] proposed that copper is stored in lysosomes in intestinal cells based on the localization of the copper dye CF4 to lysosomes and the localization of the copper transporter CUA-1.1 to lysosomes in copper excess in *C. elegans*. Here we confirmed these findings with the copper dye CF4, and we extended this analysis by performing XFM on isolated lysosomes. We detected 8.6*10^5^ and 1.1*10^6^ copper atoms per lysosome in replete and excess zinc medium, respectively. In addition, excess copper caused lysosomal remodeling characterized by an increase in the volume of the expansion compartment. This result suggests that a copper transporter is delivered to the expansion compartment by vesicle fusion. To evaluate the physiological significance of copper storage in lysosomes, we analyzed *glo-1(zu391)* mutants with a reduced number of lysosomes. These mutants were hypersensitive to both copper excess and deficiency, suggesting that copper sequestration in lysosomes stores excess copper, and copper is mobilized from lysosomes during copper deficiency. These results raise an important conceptual question—do the same lysosomes store both copper and zinc, or are these metals stored in separate lysosomes? To address this question, we performed colocalization studies of the zinc transporter CDF-2 and the copper transporter CUA-1.1. In copper replete medium, CUA-1.1 is localized primarily to the basolateral membrane of intestinal cells, whereas CDF-2 is constitutively localized to the membrane of lysosomes. Thus, in these conditions the two transporters display minimal colocalization. However, in copper excess conditions, CUA-1.1 and CDF-2 colocalize to the same lysosomes, which appear to be essentially all the LysoTracker positive lysosomes in the intestinal cells. Furthermore, CUA-1.1 and CDF-2 colocalize to both the expansion compartment and the acidified compartment. These results indicate that all lysosomes in intestinal cells store both copper and zinc. We speculate that the expansion compartment increases in volume in excess copper due to the fusion of vesicles that contain CUA-1.1. These vesicles likely originate from the basolateral plasma membrane, where CUA-1.1 is localized in copper replete conditions. Roh et al. [19] reported that PGP-2 protein localizes to the expansion compartment, so CUA-1.1 is the third protein reported to localize to the expansion compartment. Indeed, earlier work combining fluorescent copper probes and XFM reveal copper fluxes in neurons consistent with vesicle transport [57]. Recent studies are also beginning to demonstrate the emerging role of lysosomes in copper homeostasis. For example, mammalian studies have recently shown that lysosomal solute carrier 46a3(SLC56A3) mediates copper homeostasis in the liver [58]. Furthermore, copper homeostasis also seems to be influential in the response against pathogens by utilizing copper transporter ATP7A to take down phagosomes [59].

Exposure to a battery of transition metals suggests that there is morphological diversity in remodeling. Zinc exposure leads to lysosomes that display an acidified compartment that projects outward and an expansion compartment that is extremely extended (Fig. 1). Exposure to manganese and copper revealed morphological changes in which an acidified compartment appeared encapsulated within the expansion compartment. Lysosomes exposed to cadmium showed an increase in the expansion compartment and a decrease of the acidified compartment. These observations indicate that lysosomes may be an important site for stress response but also a potential storage place for toxic metals. Transition metal exposure seems to yield different spatial relationships between compartments. These results lead us to speculate that other metal ion transporters contribute to these morphological changes and ask what their counter ions could be. Furthermore, it is unclear if these morphological changes are representative of a “stable” state of a lysosome or if the morphological shifts represent a “transient” stage as we do not yet know if/how they are reversible.

Our discovery of metal-responsive expansion compartments raises questions about their evolutionary conservation. Recently, Tavakoli et al. [60] identified similar structures termed “hemifusomes” in human cells, and they propose that hemifusomes are an intermediate step toward the development of multivesicular bodies. While lysosomes are already known to undergo morphological remodeling including tubulation and spherical remodeling [61, 62], our work shows that a distinct expansion compartment has a function in transition metal homeostasis and represents a novel biological role for lysosomes that leverages a compartment. Based on our findings, we developed a model that describes copper and zinc management by lysosomes (Fig. 7). The expansion compartment is always present, but it is contracted and difficult to visualize in standard medium. In copper replete medium, CUA-1.1 is localized to the basolateral membrane, whereas CDF-2 is localized to the lysosomes. As the organism transitions to copper excess, CUA-1.1 containing vesicles depart the basolateral membrane and fuse with the expansion compartment, causing it to increase in volume and increase the capacity for copper storage in the lysosomes. Increased copper storage in lysosomes reduces the cytosolic concentration of copper. CDF-2 and CUA-1.1 reside on the same lysosomes, which store both copper and zinc. Our results raise the possibility that many metals, and perhaps some organic molecules, can be stored and released from the same lysosomes through the parallel action of specific transporters.

**Figure 7:**
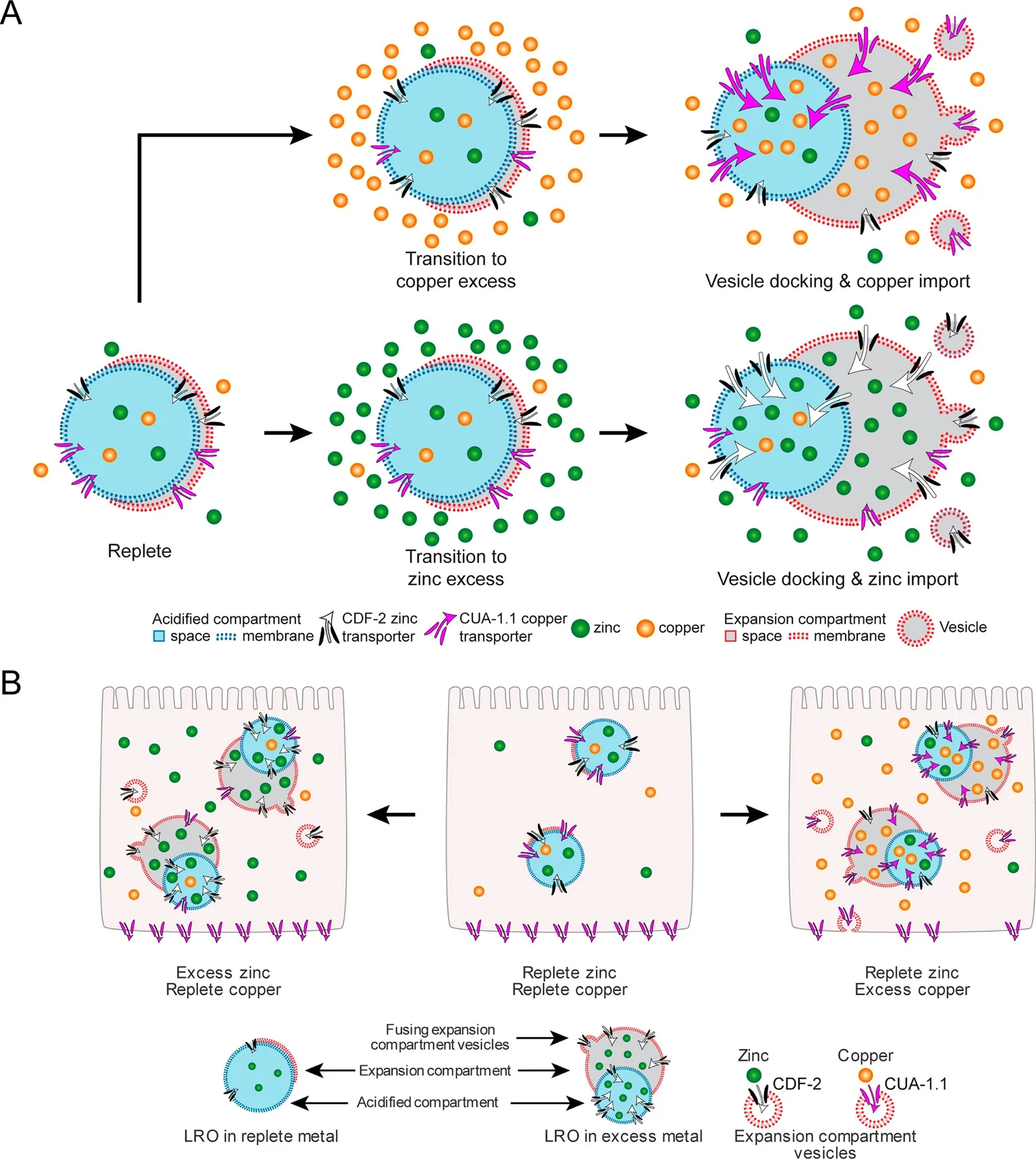
A model of lysosome remodeling during excess zinc and copper. (A) In replete medium, the lysosome has a high-volume acidified compartment (blue), a low-volume expansion compartment (red), and a small number of zinc and copper transporters. In excess copper (orange balls), vesicles containing the copper transporter CUA-1.1 (magenta) fuse to the expansion compartment, increasing the volume, number of copper transporters, and the capacity for copper storage. In excess zinc (green balls), vesicles containing the zinc transporter CDF-2 (white) fuse to the expansion compartment, increasing the volume, number of zinc transporters, and the capacity for zinc storage. While both cases lead to an increase in the volume of the expansion compartment, the transporter delivered by the expansion compartment vesicles is different. (B) In replete medium (center), an intestinal cell (pink) contains lysosomes with a low-volume expansion compartment. The copper transporter CUA-1.1 is primarily localized to the basolateral membrane, whereas the zinc transporter CDF-2 is primarily localized to the lysosome membrane. In excess zinc (left), expression of *cdf-2* mRNA and protein increases, and expansion compartment vesicles containing CDF-2 fuse to the expansion compartment to increase the volume and capacity for zinc storage. In excess copper (right), expansion compartment vesicles transfer CUA-1.1 from the basolateral plasma membrane to the lysosome membrane, increasing the volume of the expansion compartment and the capacity for copper storage.

### Experimental procedures

#### *Caenorhabditis elegans* strains

*Caenorhabditis elegans strains* were cultured on nematode growth medium dishes at 20°C with OP50 *E. coli* unless otherwise stated. To manipulate metal concentrations, we used Noble Agar Minimal Media (NAMM) because it limits precipitation of metals [63]. The wild-type strain and parent of all mutant strains was Bristol N2 [64]. Loss-of-function mutations were *cdf-2(tm788)* [18] *and glo-1(zu391)* X [34]. Extrachromosomal or integrated arrays were used to tag CDF-2, ZIPT-2.3, and CUA-1.1:*amEx342* is a multicopy extrachromosomal array that expresses CDF-2 fused to mCherry under the control of the *cdf-2* promoter (*cdf-2::mCherry; rol-6^D^*) [19], *amIs4* is an integrated multicopy array that expresses CDF-2 fused to GFP under the control of the *cdf-2* promoter (*cdf-2(tm788); amIs4)* [18]. *amEx193* is an extrachromosomal array that expresses ZIPT-2.3 fused to mCherry under the control of the *zipt-2.3* promoter [20]. To visualize CUA-1.1, we used a transgenic strain that expresses CUA-1.1 fused to GFP under the control of the *vha-6* promoter (P*_vha-6_*::CUA-1.1::GFP::unc-54 3′ UTR) [31]. The following strains were used: WU1984 contains *cdf-2(tm788); amIs4; amEx193; Pmyo2::mCherry::unc-54utr* [20]. BK014 contains P*_vha-6_*::CUA-1.1::GFP::unc-54 3′ UTR [31]. WU2132 contains *cdf-2 amEx342rol^D^*; P*_vha-6_*::CUA-1.1::GFP::unc-54 3′ UTR. WU2132 was created by crossing parental *cdf-2 amEx342rol^D^*hermaphrodites with male P*_vha-6_*::CUA-1.1::GFP::unc-54 3’ UTR worms. Worms containing both *cdf-2 amEx342rol^D^*and P*_vha-6_*::CUA-1.1::GFP::unc-54 3′ UTR were tracked across three generations.

### Transition metal and chelator supplementation

Metals and metal chelators were all dissolved in MilliQ H_2_O or ethanol. Stock solutions of metals were added to molten NAMM medium to yield a final concentration of 10−200 μM supplemental zinc (ZnSO_4_, Sigma Aldrich), 10−200 μM supplemental copper (CuCl_2_, Sigma Aldrich), 5−20 mM supplemental manganese (MnCl_2_, Sigma Aldrich), or 200 μM cadmium (CdCl_2_ Sigma Aldrich). Stock solutions of chelators were added to molten NAMM medium to yield a final concentration of 10−200 μM of the copper chelator Neocuproine (Sigma Aldrich), 10−40 μM of the zinc chelator N, N, N', N'-tetrakis(2-pyridylmethyl)ethane-1,2-diamine (TPEN, Sigma Aldrich) or 10−200 μM of the manganese chelator 1,2-Diaminocyclohexanetetraacetic acid monohydrate (DCTA, Sigma Aldrich).

### Worm growth experiments

Synchronized wild type and *glo-1(zu391)* L4 stage worms were transferred to control NAMM dishes or NAMM dishes that were supplemented with the copper chelator Neocuproine, the zinc chelator TPEN, the manganese chelator DCTA, CuCl_2_, ZnSO_4_, or MnCl_2_. Worms were cultured for 24 h on dishes seeded with *E. coli* OP50. Dishes were placed on a Leica stereomicroscope, and images of individual worms were captured. Images were analyzed for individual worm lengths using FIJI (NIH) software by measuring from the tip of the nose to the tip of the tail for each worm.

### Super-resolution confocal microscopy

L4 stage animals expressing CDF-2::GFP and ZIPT-2.3::mCherry, or CDF-2::mCherry and CUA-1.1::GFP, were cultured for 24 hours on NAMM dishes containing 200 μM zinc, 100 μM copper, 20 mM manganese, or 200 μM cadmium. To visualize copper, we diluted the copper dye CF4 into *E. coli* OP50 to obtain a final concentration of 25 μM [46]. To visualize lysosomes, we diluted LysoTracker Blue DND-22 dye (Thermofisher) in *E. coli* OP50 to a final concentration of 1 μM. Worms consumed these dyes with their bacterial food. After 24 h, the worms were picked into 50 mM NaN_3_ in MilliQ water that was pipetted onto an agar pad. The agar pad was sealed with Vaseline to a coverslip. Super-resolution microscopy was performed with the Zeiss LSM 880 II Confocal with Airyscan. Individual lysosomes were selected for analysis if they displayed all relevant signals being tested in each experiment, and they were isolated from neighboring lysosomes. Lysosome images were captured in z-stack using the 60× objective. CDF-2::GFP and CUA-1.1::GFP were detected using the 488 nm laser, LysoTracker Blue DND dye was detected using the 405 nm laser, and ZIPT-2.3::mCherry, CDF-2::mCherry and CF4 dye were detected using the 561 nm laser. Images were deconvolved using AiryScan processing to achieve ∼120 nm resolution.

### Image analysis

#### Line scans

Line scans were performed on images of lysosomes from transgenic worms expressing CDF::GFP and ZIPT-2.3::mCherry, CDF-2::GFP, CUA-1.1::GFP, and CDF-2::mCherry and CUA-1.1::GFP. Lysosomes were selected for analysis if the z-stack encompassed the entire lysosome, and if they displayed GFP, mCherry, and LysoTracker (Figs 1 and 6) or GFP, LysoTracker, and CF4 (Fig. 5). In FIJI, lines were drawn across a single center slice of a whole lysosome starting from the acidified compartment and proceeding through the expansion compartment. For control lysosomes that have a small expansion compartment, the orientation was chosen at random. The intensity of GFP, mCherry, CF4, and LysoTracker was plotted as fluorescence intensity on the *y* axis. The line drawn across the lysosome forms the *x* axis, displaying distance. For each channel, the maximum fluorescence intensity is set to 1.0 in arbitrary units (AU), and the lowest fluorescence intensity is set to 0. All other values are normalized.

Measurements of fluorescence intensity for CF4 (Fig. S3) were done in FIJI. An ROI was drawn around individual lysosomes, and the average fluorescence intensity of the ROI was reported in AU.

#### Volume calculation

The diameter (d) of each expansion and acidified compartment was measured in FIJI by drawing a line across each compartment three times and calculating the average value. In control, supplemental zinc, supplemental copper, and supplemental manganese, compartments were assumed to be spherical. Based on this assumption and the measured diameters, we calculated the radius as *r=d/2* for each compartment and then calculated the volume as $V=4/3 \pi r^{3}$. In control, supplemental zinc, supplemental copper, and supplemental manganese, the acidified compartment appears to be contained within the expansion compartment (a sphere within a sphere). To calculate the volume of the expansion compartment alone, we subtracted the volume of the acidified compartment from the volume of the total lysosome (${\rm V}_{\rm E}={\rm V}_{\rm T}-{\rm V}_{\rm A}$). For the cadmium exposure experiments, the expansion compartment appears to have the shape of a cap on a sphere instead of a sphere itself. Therefore, we calculated the total volume of the lysosome by measuring the diameter three times and then calculating the average value. The total volume of the sphere was calculated as $V=4/3 \pi r^{3}$. To determine the volume of the expansion compartment, we measured the volume of the cap on the sphere with the equation $V=\frac{\pi h^{2}}{3}(3r-h)$; we measured r three times to determine the average. To determine the volume of the acidified compartment, we subtracted the expansion compartment volume from the total volume (${\rm V}_{\rm A}={\rm V}_{\rm T}-{\rm V}_{\rm E}$).

### XFM sample preparation, scanning parameters, and data analysis

#### Preparation of isolated lysosomes

L4 stage worms were picked from dishes with mixed populations and cultured on control and zinc supplemented (200 μM ZnSO_4_) NAMM dishes for 24 h, where they develop into young adults. A microscope slide was set up to accommodate three drops of Milli-Q water and one drop of 100 mM ammonium acetate buffer. None of the drops were allowed to touch each other to avoid contamination. Using a stereomicroscope, worms were successively transferred by picking into each water drop to wash off bacteria and then transferred by picking into the drop of ammonium acetate buffer. While in the ammonium acetate buffer, worms were cut with a needle to extrude the intestine. Lysosomes near the edge of the cut were allowed to freely flow into the buffer. The isolated lysosomes were subsequently pipetted and transferred to a Silicon Nitride window (Silson) using a 150 μm oocyte striper tip (Cooper Surgical) and allowed to air dry.

#### XFM

Isolated lysosomes were raster scanned with a pixel size of 80 nm and a dwell time of 500 ms per pixel. A double-crystal Si < 111 > monochromator and a pair of Fresnel zone plates were used to deliver a 10 keV focused beam of ∼80 nm. All XFM experiments were performed at the Bionanoprobe [65] at the Advanced Photon Source (Argonne National Laboratory, Argonne IL).

#### Data analysis

All data were analyzed using the MAPS software [66]. ROIs of individual lysosomes were traced in MAPS, and quantities obtained from whole individual lysosomes were generated in MAPS in femtograms. To calculate the total elemental content for each ROI, we subtracted the background from the initial femtograms to generate the background subtracted mass. The mass was then converted to atoms/lysosome.

### Statistical analysis

Statistical tests for Fig. 1. (A). One-Way Anova with post hoc test to account for non-normal distribution of replete, copper, and manganese conditions. (C). One-Way Anova with post hoc test to account for non-normal distribution of replete, copper, and cadmium conditions. (D). One-Way Anova with post hoc test to account for non-normal distribution of copper and cadmium conditions. (E). One-Way Anova with post hoc test to account for non-normal distribution of zinc, copper, and manganese conditions. (F). One-Way Anova with post hoc test to account for non-normal distribution of copper and cadmium conditions.

Statistical tests for Fig. 2. (A). For analysis of individual concentrations between WT and *glo-1(lf)* mutants a two-way Anova was performed with a post hoc test to account for 10 μM TPEN and 10 μm zinc. (B). For analysis of individual concentrations between WT and *glo-1(lf)* mutants a two-way Anova was performed with a post hoc test to account for non-normally distributed 25 μM Neocuproine and 25 μM copper. (C). For analysis of individual concentrations between WT and *glo-1(lf)* mutants a two-way Anova was performed with a post hoc test to account for non-normally distributed (200, 150, 100, 25, 10, and 0 μM DCTA and 5 mM manganese).

Statistical test for Fig. 4 (K), Fig. S2 (A−I), and Fig. S3 (C) Mann Whitney t-test to account for non-normal distribution of data.

Statistical test for Fig. S4 conditions CDF-2 (A, C, E) Mann Whitney t-test to account for non-normal distribution of data (D, F) Welch’s t-test to account for normal distribution. Condition CUA-1.1 (D, E, F) Mann Whitney t-test to account for non-normal distribution of data (A, C) Welch’s t-test to account for normal distribution.

Statistical test for Fig. S5 (A, C, E) Mann Whitney t-test to account for non-normal distribution of data. (D, F) Welch’s t-test to account for normal distribution.

*P* value < .05 was considered statistically significant.

## Supplementary Material

mfag030_Supplemental_Files

## Data Availability

The data underlying this article are available in the article and in its online Supplementary material.
