# Supplementary material for: Lysosomal expansion compartments mediate zinc and copper homeostasis in Caenorhabditis elegans
Source: Metallomics. 2026 Oct 7;18(1):mfag030. doi: 10.1093/mtomcs/mfag030 (PMC13692797; doi:10.1093/mtomcs/mfag030)
Supplement: Supplementary file 1 [file mfag030_supplemental_files.zip › Supplemental Table1.docx]

**Table S1: Individual lysosome values of Figure 1 graphs by condition**

| Control |  |  |  |  |  |
| --- | --- | --- | --- | --- | --- |
| Lysosome # | Total Volume (μm^3^) | Acidified  Compartment  Volume (μm^3^) | Expansion Compartment  Volume (μm^3^) | Acidified Compartment (% of total) | Expansion Compartment  (% of total) |
| 1 | 1.37 | 0.988 | 0.380 | 72 | 28 |
| 2 | 3.24 | 2.81 | 0.435 | 87 | 13 |
| 3 | 1.55 | 0.608 | 0.938 | 39 | 61 |
| 4 | 1.72 | 1.14 | 0.586 | 66 | 34 |
| 5 | 1.51 | 1.12 | 0.397 | 74 | 26 |
| 6 | 3.57 | 3.57 | 0.000 | 100 | 0 |
| 7 | 2.02 | 1.16 | 0.855 | 58 | 42 |
| 8 | 1.38 | 0.505 | 0.876 | 37 | 63 |
| 9 | 2.12 | 0.798 | 1.32 | 38 | 62 |
| 10 | 5.14 | 4.04 | 1.09 | 79 | 21 |
| 11 | 1.54 | 0.887 | 0.649 | 58 | 42 |
| 12 | 0.43 | 0.406 | 0.022 | 95 | 5 |
| 13 | 2.85 | 1.09 | 1.76 | 38 | 62 |
| 14 | 1.43 | 0.885 | 0.540 | 62 | 38 |
| 15 | 2.93 | 1.77 | 1.16 | 60 | 40 |
| 16 | 1.89 | 0.854 | 1.03 | 45 | 55 |
| 17 | 2.00 | 0.448 | 1.56 | 22 | 78 |
| 18 | 1.59 | 0.549 | 1.04 | 34 | 66 |
| 19 | 1.76 | 0.925 | 0.831 | 53 | 47 |
| 20 | 3.28 | 2.29 | 0.995 | 70 | 30 |
| 21 | 15.1 | 15.1 | 0.000 | 100 | 0 |
| 22 | 14.6 | 14.6 | 0.000 | 100 | 0 |
| 23 | 14.9 | 13.9 | 0.982 | 93 | 7 |
| 24 | 14.4 | 14.4 | 0.00 | 100 | 0 |
| 25 | 5.58 | 4.83 | 0.752 | 87 | 13 |
|  |  |  |  |  |  |
| Averages | 4.31 | 3.59 | 0.728 | 67 | 33 |
|  |  |  |  |  |  |
| Zinc |  |  |  |  |  |
| Lysosome # | Total Volume (μm^3^) | Acidified Compartment  Volume (μm^3^) | Expansion Compartment  Volume (μm^3^) | Acidified Compartment (% of total) | Expansion Compartment (% of total) |
| 1 | 20.5 | 3.35 | 17.1 | 16 | 84 |
| 2 | 19.2 | 4.29 | 14.9 | 22 | 78 |
| 3 | 19.9 | 3.90 | 16.0 | 20 | 80 |
| 4 | 6.40 | 1.57 | 4.83 | 25 | 75 |
| 5 | 2.49 | 0.606 | 1.89 | 24 | 76 |
| 6 | 9.66 | 1.09 | 8.57 | 11 | 89 |
| 7 | 12.4 | 2.17 | 10.2 | 18 | 82 |
| 8 | 24.4 | 3.78 | 20.6 | 15 | 85 |
| 9 | 10.8 | 3.54 | 7.26 | 33 | 67 |
| 10 | 26.4 | 6.42 | 20.0 | 24 | 76 |
| 11 | 7.84 | 1.38 | 6.46 | 18 | 82 |
| 12 | 14.6 | 2.32 | 12.3 | 16 | 84 |
| 13 | 9.18 | 1.25 | 7.93 | 14 | 86 |
| 14 | 17.5 | 1.91 | 15.6 | 11 | 89 |
| 15 | 14.8 | 0.840 | 13.9 | 6 | 94 |
| 16 | 21.3 | 3.33 | 18.0 | 16 | 84 |
| 17 | 6.66 | 0.73 | 5.93 | 11 | 89 |
| 18 | 9.06 | 2.41 | 6.65 | 27 | 73 |
| 19 | 18.5 | 3.71 | 14.8 | 20 | 80 |
| 20 | 41.4 | 2.48 | 38.9 | 6 | 94 |
| 21 | 44.3 | 3.09 | 41.2 | 7 | 93 |
| 22 | 42.8 | 2.96 | 39.8 | 7 | 93 |
|  |  |  |  |  |  |
| Averages | 18.2 | 2.60 | 15.6 | 17 | 83 |
|  |  |  |  |  |  |
| Copper |  |  |  |  |  |
| Lysosome # | Total Volume (μm^3^) | Acidified Compartment  Volume (μm^3^) | Expansion Compartment  Volume (μm^3^) | Acidified Compartment (% of total) | Expansion Compartment  (% of total) |
| 1 | 7.00 | 0.154 | 6.85 | 2 | 98 |
| 2 | 9.25 | 3.49 | 5.76 | 38 | 62 |
| 3 | 17.0 | 0.728 | 16.2 | 4 | 96 |
| 4 | 11.0 | 1.35 | 9.68 | 12 | 88 |
| 5 | 13.3 | 0.453 | 12.8 | 3 | 97 |
| 6 | 10.9 | 1.35 | 9.53 | 12 | 88 |
| 7 | 5.81 | 1.38 | 4.43 | 24 | 76 |
| 8 | 7.51 | 0.665 | 6.84 | 9 | 91 |
| 9 | 5.53 | 0.343 | 5.19 | 6 | 94 |
| 10 | 6.57 | 3.28 | 3.30 | 50 | 50 |
| 11 | 4.70 | 4.19 | 0.510 | 89 | 11 |
| 12 | 7.38 | 2.57 | 4.81 | 35 | 65 |
| 13 | 3.94 | 0.983 | 2.96 | 25 | 75 |
| 14 | 4.10 | 2.12 | 1.98 | 52 | 48 |
| 15 | 6.98 | 1.58 | 5.40 | 23 | 77 |
| 16 | 7.90 | 1.77 | 6.13 | 22 | 78 |
| 17 | 3.32 | 1.04 | 2.28 | 31 | 69 |
| 18 | 3.19 | 2.74 | 0.45 | 86 | 14 |
| 19 | 2.75 | 1.88 | 0.87 | 68 | 32 |
| 20 | 2.33 | 0.388 | 1.95 | 17 | 83 |
| 21 | 49.4 | 7.77 | 41.6 | 16 | 84 |
| 22 | 45.1 | 3.32 | 41.8 | 7 | 93 |
| 23 | 21.3 | 3.28 | 18.0 | 15 | 85 |
| 24 | 38.1 | 5.16 | 32.9 | 14 | 86 |
|  |  |  |  |  |  |
| Averages | 12.3 | 2.17 | 10.1 | 27.5 | 72.5 |
|  |  |  |  |  |  |
| Manganese |  |  |  |  |  |
| Lysosome # | Total Volume (μm^3^) | Acidified Compartment  Volume (μm^3^) | Expansion Compartment  Volume (μm^3^) | Acidified Compartment (% of total) | Expansion Compartment (% of total) |
| 1 | 15.6 | 7.32 | 8.27 | 47 | 53 |
| 2 | 9.88 | 5.69 | 4.18 | 58 | 42 |
| 3 | 7.66 | 3.85 | 3.80 | 50 | 50 |
| 4 | 19.1 | 5.06 | 14.0 | 26 | 74 |
| 5 | 15.1 | 7.36 | 7.73 | 49 | 51 |
| 6 | 15.7 | 4.37 | 11.3 | 28 | 72 |
| 7 | 6.69 | 5.68 | 1.01 | 85 | 15 |
| 8 | 11.7 | 7.55 | 4.13 | 65 | 35 |
| 9 | 5.36 | 5.36 | 0 | 100 | 0 |
| 10 | 10.2 | 7.82 | 2.38 | 77 | 23 |
| 11 | 16.4 | 9.68 | 6.76 | 59 | 41 |
| 12 | 10.1 | 6.30 | 3.77 | 63 | 37 |
| 13 | 3.42 | 1.39 | 2.03 | 41 | 59 |
| 14 | 6.73 | 6.73 | 0 | 100 | 0 |
| 15 | 14.9 | 14.0 | 0.913 | 94 | 6 |
| 16 | 13.4 | 5.75 | 7.69 | 43 | 57 |
| 17 | 7.17 | 3.52 | 3.66 | 49 | 51 |
| 18 | 6.82 | 4.10 | 2.71 | 60 | 40 |
| 19 | 6.89 | 3.15 | 3.73 | 46 | 54 |
| 20 | 7.13 | 3.04 | 4.09 | 43 | 57 |
| 21 | 39.8 | 8.71 | 31.1 | 22 | 78 |
| 22 | 21.5 | 6.55 | 15.0 | 30 | 70 |
| 23 | 17.7 | 1.28 | 16.4 | 7 | 93 |
| 24 | 9.60 | 2.74 | 6.86 | 29 | 71 |
| 25 | 31.0 | 0.552 | 30.5 | 2 | 98 |
|  |  |  |  |  |  |
| Averages | 13.2 | 5.50 | 7.68 | 51 | 49 |
|  |  |  |  |  |  |
| Cadmium |  |  |  |  |  |
| Lysosome # | Total Volume (μm^3^) | Acidified Compartment Volume (μm^3^) | Expansion Compartment Volume (μm^3^) | Acidified Compartment (% of total) | Expansion Compartment (% of total) |
| 1 | 1.86 | 0.351 | 1.51 | 19 | 81 |
| 2 | 1.12 | 0.532 | 0.591 | 47 | 53 |
| 3 | 4.07 | 2.91 | 1.16 | 71 | 29 |
| 4 | 4.40 | 4.20 | 0.207 | 95 | 5 |
| 5 | 6.59 | 1.44 | 5.15 | 22 | 78 |
| 6 | 1.45 | 0.389 | 1.07 | 27 | 73 |
| 7 | 6.47 | 2.59 | 3.89 | 40 | 60 |
| 8 | 7.75 | 3.12 | 4.63 | 40 | 60 |
| 9 | 9.24 | 3.87 | 5.37 | 42 | 58 |
| 10 | 6.57 | 0.576 | 5.99 | 9 | 91 |
| 11 | 6.01 | 3.77 | 2.24 | 63 | 37 |
| 12 | 7.24 | 7.24 | 0.000 | 100 | 0 |
| 13 | 5.90 | 0.399 | 5.50 | 7 | 93 |
| 14 | 12.5 | 1.95 | 10.6 | 16 | 84 |
| 15 | 8.93 | 1.91 | 7.01 | 21 | 79 |
| 16 | 12.2 | 3.01 | 9.24 | 25 | 75 |
| 17 | 8.11 | 2.51 | 5.60 | 31 | 69 |
| 18 | 7.03 | 1.93 | 5.10 | 27 | 73 |
| 19 | 4.66 | 1.54 | 3.12 | 33 | 67 |
| 20 | 8.65 | 2.55 | 6.10 | 29 | 71 |
|  |  |  |  |  |  |
| Averages | 6.54 | 2.34 | 4.20 | 38 | 62 |
