# Supplementary material for: Lysosomal expansion compartments mediate zinc and copper homeostasis in Caenorhabditis elegans
Source: Metallomics. 2026 Oct 7;18(1):mfag030. doi: 10.1093/mtomcs/mfag030 (PMC13692797; doi:10.1093/mtomcs/mfag030)
Supplement: Supplementary file 1 [file mfag030_supplemental_files.zip › Supplemental Table2.docx]

**Table S2: Individual lysosome values of Figure 5 graphs by condition**

| Replete- CDF-2 | |  |  |  |  |
| --- | --- | --- | --- | --- | --- |
| Lysosome # | Total Volume (μm^3^) | Acidified Compartment Volume (μm^3^) | Expansion Compartment Volume (μm^3^) | Acidified Compartment  (% of total) | Expansion Compartment (% of total) |
| 1 | 2.10 | 2.10 | 0.000 | 100 | 0 |
| 2 | 1.21 | 1.05 | 0.160 | 87 | 13 |
| 3 | 2.28 | 1.62 | 0.659 | 71 | 29 |
| 4 | 1.61 | 1.30 | 0.309 | 81 | 19 |
| 5 | 3.12 | 2.08 | 1.04 | 67 | 33 |
| 6 | 1.57 | 1.42 | 0.153 | 90 | 10 |
| 7 | 6.06 | 5.05 | 1.01 | 83 | 17 |
| 8 | 6.30 | 4.88 | 1.43 | 77 | 23 |
| 9 | 7.39 | 7.39 | 0.000 | 100 | 0 |
| 10 | 7.09 | 6.24 | 0.847 | 88 | 12 |
| 11 | 2.81 | 2.77 | 0.035 | 99 | 1 |
| 12 | 5.18 | 4.97 | 0.207 | 96 | 4 |
| 13 | 8.75 | 8.75 | 0.000 | 100 | 0 |
| 14 | 7.89 | 7.22 | 0.667 | 92 | 8 |
| 15 | 7.93 | 7.93 | 0.000 | 100 | 0 |
| 16 | 2.89 | 2.89 | 0.000 | 100 | 0 |
| 17 | 5.07 | 3.42 | 1.64 | 68 | 32 |
| 18 | 6.48 | 5.56 | 0.927 | 86 | 14 |
| 19 | 5.66 | 3.26 | 2.40 | 58 | 42 |
| 20 | 3.38 | 3.38 | 0.000 | 100 | 0 |
|  |  |  |  |  |  |
| Averages | 4.74 | 4.16 | 0.575 | 87 | 13 |
|  |  |  |  |  |  |
| Copper CDF-2 | |  |  |  |  |
| Lysosome # | Total Volume (μm^3^) | Acidified Compartment Volume (μm^3^) | Expansion Compartment  Volume (μm^3^) | Acidified Compartment (% of total) | Expansion Compartment (% of total) |
| 1 | 6.73 | 3.53 | 3.20 | 52 | 48 |
| 2 | 3.24 | 0.905 | 2.33 | 28 | 72 |
| 3 | 4.59 | 1.28 | 3.31 | 28 | 72 |
| 4 | 2.43 | 0.443 | 1.99 | 18 | 82 |
| 5 | 1.71 | 0.730 | 0.981 | 43 | 57 |
| 6 | 4.82 | 1.81 | 3.01 | 38 | 62 |
| 7 | 1.95 | 0.812 | 1.13 | 42 | 58 |
| 8 | 4.90 | 1.24 | 3.66 | 25 | 75 |
| 9 | 3.24 | 1.22 | 2.03 | 38 | 62 |
| 10 | 4.42 | 1.44 | 2.98 | 33 | 67 |
| 11 | 4.62 | 1.29 | 3.33 | 28 | 72 |
| 12 | 4.51 | 1.36 | 3.15 | 30 | 70 |
| 13 | 2.85 | 0.767 | 2.08 | 27 | 73 |
| 14 | 4.64 | 0.965 | 3.67 | 21 | 79 |
| 15 | 7.66 | 2.90 | 4.76 | 38 | 62 |
| 16 | 5.27 | 2.26 | 3.01 | 43 | 57 |
| 17 | 2.53 | 1.09 | 1.44 | 43 | 57 |
| 18 | 3.46 | 1.11 | 2.35 | 32 | 68 |
| 19 | 3.79 | 1.43 | 2.36 | 38 | 62 |
| 20 | 4.78 | 1.76 | 3.02 | 37 | 63 |
|  |  |  |  |  |  |
| Averages | 4.11 | 1.42 | 2.69 | 34 | 66 |
|  |  |  |  |  |  |
| Replete- CUA-1.1 | |  |  |  |  |
| Lysosome # | Total Volume (μm^3^) | Acidified Compartment Volume (μm^3^) | Expansion Compartment Volume (μm^3^) | Acidified Compartment (% of total) | Expansion Compartment (% of total) |
| 1 | 1.18 | 1.18 | 0.000 | 100 | 0 |
| 2 | 2.97 | 2.51 | 0.464 | 84 | 16 |
| 3 | 3.35 | 3.33 | 0.013 | 99 | 1 |
| 4 | 1.76 | 1.66 | 0.097 | 94 | 6 |
| 5 | 3.18 | 2.88 | 0.297 | 91 | 9 |
| 6 | 2.30 | 2.30 | 0.000 | 100 | 0 |
| 7 | 3.68 | 3.68 | 0.000 | 100 | 0 |
| 8 | 3.19 | 3.19 | 0.000 | 100 | 0 |
| 9 | 1.20 | 1.20 | 0.000 | 100 | 0 |
| 10 | 4.01 | 3.91 | 0.093 | 98 | 2 |
| 11 | 2.85 | 2.85 | 0.000 | 100 | 0 |
| 12 | 1.43 | 1.43 | 0.000 | 100 | 0 |
| 13 | 3.27 | 3.27 | 0.000 | 100 | 0 |
| 14 | 4.33 | 4.33 | 0.000 | 100 | 0 |
| 15 | 1.03 | 1.03 | 0.000 | 100 | 0 |
| 16 | 2.33 | 2.33 | 0.000 | 100 | 0 |
| 17 | 0.617 | 0.527 | 0.089 | 86 | 14 |
| 18 | 0.436 | 0.404 | 0.032 | 93 | 7 |
| 19 | 0.338 | 0.236 | 0.102 | 70 | 30 |
| 20 | 1.06 | 0.485 | 0.577 | 46 | 54 |
|  |  |  |  |  |  |
| Averages | 2.23 | 2.14 | 0.088 | 93 | 7 |
|  |  |  |  |  |  |
| Copper- CUA-1.1 | |  |  |  |  |
| Lysosome # | Total Volume (μm^3^) | Acidified Compartment  Volume (μm^3^) | Expansion Compartment Volume (μm^3^) | Acidified Compartment (% of total) | Expansion Compartment (% of total) |
| 1 | 24.2 | 5.54 | 18.6 | 23 | 77 |
| 2 | 7.45 | 2.28 | 5.17 | 31 | 69 |
| 3 | 15.4 | 5.15 | 10.3 | 33 | 67 |
| 4 | 13.0 | 5.65 | 7.39 | 43 | 57 |
| 5 | 20.3 | 7.34 | 12.9 | 36 | 64 |
| 6 | 15.8 | 4.47 | 11.3 | 28 | 72 |
| 7 | 19.2 | 7.83 | 11.4 | 41 | 59 |
| 8 | 12.1 | 3.14 | 8.99 | 26 | 74 |
| 9 | 9.37 | 1.33 | 8.05 | 14 | 86 |
| 10 | 7.44 | 2.25 | 5.19 | 30 | 70 |
| 11 | 4.83 | 0.994 | 3.84 | 21 | 79 |
| 12 | 6.84 | 1.56 | 5.28 | 23 | 77 |
| 13 | 5.90 | 1.47 | 4.44 | 25 | 75 |
| 14 | 19.4 | 5.93 | 13.4 | 31 | 69 |
| 15 | 3.80 | 0.690 | 3.11 | 18 | 82 |
| 16 | 22.6 | 3.28 | 19.3 | 15 | 85 |
| 17 | 7.76 | 1.33 | 6.43 | 17 | 83 |
| 18 | 8.24 | 1.00 | 7.23 | 12 | 88 |
| 19 | 4.06 | 1.02 | 3.05 | 25 | 75 |
| 20 | 12.2 | 3.00 | 9.21 | 25 | 75 |
|  |  |  |  |  |  |
| Averages | 12.0 | 3.26 | 8.73 | 26 | 74 |
