# Supplementary material for: Lysosomal expansion compartments mediate zinc and copper homeostasis in Caenorhabditis elegans
Source: Metallomics. 2026 Oct 7;18(1):mfag030. doi: 10.1093/mtomcs/mfag030 (PMC13692797; doi:10.1093/mtomcs/mfag030)
Supplement: Supplementary file 1 [file mfag030_supplemental_files.zip › Supplemental Table3.docx]

**Table S3: Individual lysosome values of Figure 6 graphs by condition**

| Replete |  |  |  |  |  |
| --- | --- | --- | --- | --- | --- |
| Lysosome # | Total Volume (μm^3^) | Acidified Compartment Volume (μm^3^) | Expansion Compartment Volume (μm^3^) | Acidified Compartment (% of total) | Expansion Compartment  (% of total) |
| 1 | 0.948 | 0.664 | 0.284 | 70 | 30 |
| 2 | 0.674 | 0.361 | 0.313 | 54 | 46 |
| 3 | 1.05 | 0.655 | 0.394 | 62 | 38 |
| 4 | 1.33 | 0.880 | 0.446 | 66 | 34 |
| 5 | 1.67 | 0.744 | 0.926 | 45 | 55 |
| 6 | 1.11 | 0.925 | 0.180 | 84 | 16 |
| 7 | 1.23 | 0.669 | 0.563 | 54 | 46 |
| 8 | 1.04 | 0.640 | 0.404 | 61 | 39 |
| 9 | 1.32 | 0.891 | 0.426 | 68 | 32 |
| 10 | 1.38 | 0.993 | 0.390 | 72 | 28 |
| 11 | 13.1 | 10.6 | 2.52 | 81 | 19 |
| 12 | 8.04 | 7.89 | 0.154 | 98 | 2 |
| 13 | 8.86 | 7.38 | 1.48 | 83 | 17 |
| 14 | 22.1 | 19.4 | 2.71 | 88 | 12 |
| 15 | 8.59 | 6.70 | 1.89 | 78 | 22 |
| 16 | 7.18 | 5.15 | 2.03 | 72 | 28 |
| 17 | 5.22 | 3.64 | 1.61 | 69 | 31 |
| 18 | 6.73 | 4.44 | 2.29 | 66 | 34 |
| 19 | 15.1 | 11.1 | 3.98 | 74 | 26 |
| 20 | 2.48 | 1.60 | 0.885 | 64 | 36 |
| 21 | 2.23 | 1.34 | 0.893 | 60 | 40 |
| 22 | 4.74 | 3.09 | 1.65 | 65 | 35 |
| 23 | 3.18 | 2.36 | 0.817 | 74 | 26 |
| 24 | 12.0 | 8.80 | 3.18 | 73 | 27 |
| 25 | 16.3 | 12.6 | 3.75 | 77 | 23 |
|  |  |  |  |  |  |
| Averages | 5.90 | 4.54 | 1.37 | 70 | 30 |
|  |  |  |  |  |  |
| Copper |  |  |  |  |  |
| Lysosome # | Total Volume (μm^3^) | Acidified Compartment  Volume (μm^3^) | Expansion Compartment Volume (μm^3^) | Acidified Compartment (% of total) | Expansion Compartment (% of total) |
| 1 | 2.30 | 1.34 | 0.964 | 58 | 42 |
| 2 | 1.76 | 1.01 | 0.750 | 57 | 43 |
| 3 | 2.31 | 0.690 | 1.62 | 30 | 70 |
| 4 | 9.26 | 2.94 | 6.32 | 32 | 68 |
| 5 | 6.64 | 3.15 | 3.49 | 47 | 53 |
| 6 | 2.04 | 1.30 | 0.742 | 64 | 36 |
| 7 | 3.78 | 1.48 | 2.30 | 39 | 61 |
| 8 | 1.78 | 0.503 | 1.28 | 28 | 72 |
| 9 | 1.08 | 0.296 | 0.781 | 27 | 73 |
| 10 | 2.42 | 0.634 | 1.78 | 26 | 74 |
| 11 | 2.22 | 0.765 | 1.45 | 35 | 65 |
| 12 | 1.40 | 0.112 | 1.29 | 8 | 92 |
| 13 | 1.79 | 0.159 | 1.63 | 9 | 91 |
| 14 | 0.76 | 0.215 | 0.547 | 28 | 72 |
| 15 | 1.34 | 0.287 | 1.05 | 21 | 79 |
| 16 | 1.74 | 0.148 | 1.59 | 9 | 91 |
| 17 | 2.52 | 0.476 | 2.04 | 19 | 81 |
| 18 | 1.88 | 0.581 | 1.30 | 31 | 69 |
| 19 | 3.32 | 1.15 | 2.17 | 35 | 65 |
| 20 | 8.42 | 3.53 | 4.88 | 42 | 58 |
| 21 | 3.35 | 1.25 | 2.10 | 37 | 63 |
| 22 | 1.41 | 0.286 | 1.13 | 20 | 80 |
| 23 | 5.19 | 1.82 | 3.37 | 35 | 65 |
| 24 | 5.82 | 2.48 | 3.34 | 43 | 57 |
| 25 | 2.67 | 0.967 | 1.70 | 36 | 64 |
|  |  |  |  |  |  |
| Averages | 3.09 | 1.10 | 1.99 | 33 | 67 |
